# Antimicrobial *Bacillus*: Metabolites and Their Mode of Action

**DOI:** 10.3390/antibiotics11010088

**Published:** 2022-01-12

**Authors:** Charlie Tran, Ian E. Cock, Xiaojing Chen, Yunjiang Feng

**Affiliations:** 1 Griffith Institute for Drug Discovery (GRIDD), Griffith University, Nathan, QLD 4111, Australia; charlie.tran@griffithuni.edu.au; 2 School of Environment and Science, Griffith University, Nathan, QLD 4111, Australia; i.cock@griffith.edu.au; 3 Bioproton Pty Ltd., Acacia Ridge, QLD 4110, Australia; wendy@bioproton.com

**Keywords:** antimicrobials, *Bacillus*, probiotic, animal feed, omics

## Abstract

The agricultural industry utilizes antibiotic growth promoters to promote livestock growth and health. However, the World Health Organization has raised concerns over the ongoing spread of antibiotic resistance transmission in the populace, leading to its subsequent ban in several countries, especially in the European Union. These restrictions have translated into an increase in pathogenic outbreaks in the agricultural industry, highlighting the need for an economically viable, non-toxic, and renewable alternative to antibiotics in livestock. Probiotics inhibit pathogen growth, promote a beneficial microbiota, regulate the immune response of its host, enhance feed conversion to nutrients, and form biofilms that block further infection. Commonly used lactic acid bacteria probiotics are vulnerable to the harsh conditions of the upper gastrointestinal system, leading to novel research using spore-forming bacteria from the genus *Bacillus*. However, the exact mechanisms behind *Bacillus* probiotics remain unexplored. This review tackles this issue, by reporting antimicrobial compounds produced from *Bacillus* strains, their proposed mechanisms of action, and any gaps in the mechanism studies of these compounds. Lastly, this paper explores omics approaches to clarify the mechanisms behind *Bacillus* probiotics.

## 1. Introduction

Probiotics are live microorganisms that can be consumed by its host to confer a range of health benefits. These benefits include the production of antimicrobial metabolites, restoration of the host microbiota, modulation of the immune system, and the release of digestive enzymes to improve nutrient uptake [1]. For example, *Bacillus subtilis* MA139 restored microbiota diversity in finishing pigs, improved their resistance to pathogenic illnesses, and promoted animal health and growth [2]. This increase in animal production makes probiotics a suitable alternative to antibiotic use in animals, due to the WHO advocating for its restricted use and its subsequent ban by the EU in 2006 [3].

Probiotics are commonly used in animal feed production, which do not contribute to antibiotic resistance and may even reduce it [4]. Selective probiotic bacteria have been used to treat antibiotic-associated diarrhea (AAD), a common side-effect of antibiotic use. Antibiotics elevate the risk of AAD by disrupting the diversity of the gut biota, allowing the proliferation of opportunistic pathogens such as *Clostridium difficile* [5]. This issue can be tackled through the use of probiotics, which inhibit pathogen growth and restabilize the intestinal microbiota back to normal levels [6]. Furthermore, probiotics can bind to the intestinal walls of its host and competitively exclude competing pathogens. Additionally, these probiotics produce a plethora of antimicrobial compounds that target pathogenic bacteria, which has driven the search for a potent probiotic strain for industrial use.

The issue lies in the presence of antibiotic resistance genes, with the commonly used *Lactobacillus* showing frequent resistance to vancomycin, ciprofloxacin, and aminoglycosides [7]. This development has driven the research into other probiotic genera not yet explored such as *Bacillus*. *Bacillus* probiotics are pore-forming bacteria that can survive the harsh conditions needed for pelletizing and can tolerate aerobic conditions for industrial use, unlike *Lactobacillus* and *Bifidobacterium* [8].

Several review papers have been published in the literature summarizing *Bacillus* metabolites, structural classes, and their antimicrobial activities [9,10,11]. However, no literature is available investigating the mechanisms of action of the antimicrobial metabolites from *Bacillus*. In this review, we summarized 47 antimicrobial compounds based on their molecular targets in the cell wall, plasma membrane, intracellular processes, and other emerging targets.

## 2. A Glance of Bioactive *Bacillus* and Their Antimicrobial Metabolites

**Figure 1:**
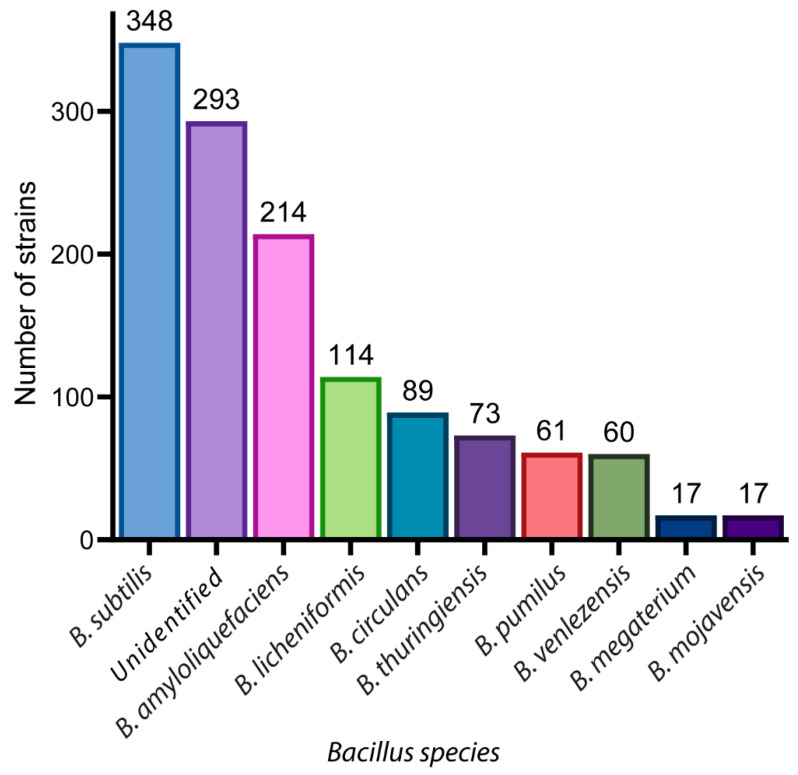
The number of *Bacillus* strains reported for each species.

To gain a good understanding of antimicrobial *Bacillus* sp., and hence their potential as a probiotic supplement, we conducted a literature review on antimicrobial Bacillus. Google-Scholar, PubMed, Scopus, and Science-Direct electronic databases were used to identify original scientific research papers. The terms ‘antimicrobial Bacillus’ and ‘mechanism of action’ were used as filters, with the earliest possible time range. Our literature search revealed that 1389 *Bacillus* strains have been reported for antimicrobial activity, composed of 27 different species (Figure 1). The most commonly reported species included *subtilis* (*n* = 348), *amyloliquefaciens* (*n* = 214), *licheniformis* (*n* = 114), *circulans* (*n* = 89), *thuringiensis* (*n* = 73), *pumilus* (*n* = 61), *velezensis* (*n* = 60), *megaterium* (*n* = 17), and *mojavensis* (*n* = 17) (Figure 1). The literature review also suggested that a substantial number of *Bacillus* species were not identified (*n* = 293). From the antimicrobial *Bacillus* sp., 47 metabolites have been identified and their mechanisms of actions reported [12]. We herein report the chemical structures of the metabolites, their antimicrobial activity, and mechanism of action. Details regarding these compounds, including source strain, anti-microbial activity, molecular target, and references are provided in Supplementary Table S1.

## 3. Antimicrobial Metabolites and Their Mechanism of Action

### 3.1. Metabolites Targeting the Cell Wall

**Figure 2:**
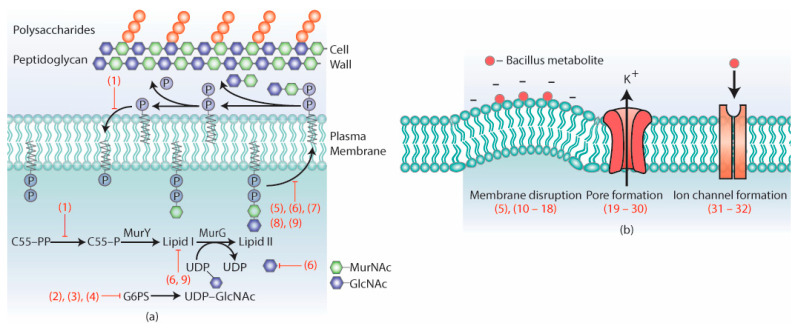
Metabolites targeting (**a**) cell wall and (**b**) plasma membrane.

The cell wall is a selectively permeable layer that has a distinct layer of polysaccharides, peptidoglycans, and fungi-specific chitins and glucans [13]. This structure is located outside the plasma membrane and acts as a permeable barrier, which regulates the entry of metabolites into the cell and protects it against external stresses (Figure 2a). The cell wall is a promising target for drug development due to its absence in mammalian cells, and several *Bacillus* strains have been shown to target this structure by releasing enzymes (amylase, cellulase, chitinase, chitosanase, glucanase, and protease) and antimicrobial metabolites. From the reported 47 compounds with clearly defined mechanisms, 9 compounds target the cell wall (Figure 2a).

**Figure 3:**
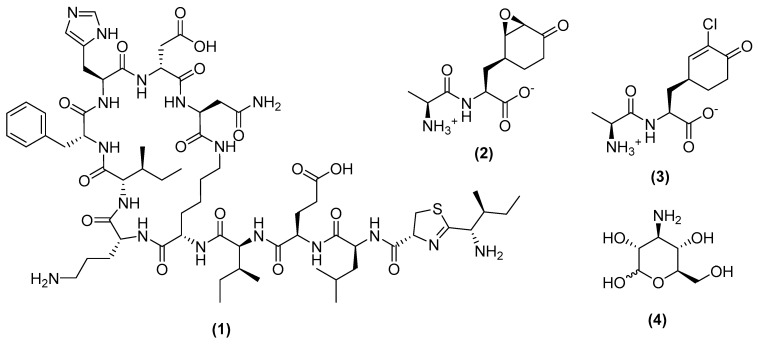
Chemical structures of bacitracin A (**1**), bacilysin (**2**), chlorotetaine (**3**), and kanosamine (**4**).

The peptidoglycan layer provides integrity and protection to the cell. This layer is comprised of linear glycan strands, which alternate between N-acetylglucosamine (GlcNAc) and N-acetylmuramic acid (MurNAc) residues linked by β-1-4 bonds [14]. Bacitracin, an antibiotic first isolated from *B. licheniformis,* primarily acts on gram-positive bacteria such as *Streptococcus mutans* (MIC = 78.12 μg/mL) [15,16]. This antibiotic is comprised of a mixture of compounds, which include bacitracin A (1), B and C. Bacitracin A (Figure 3) prevents the dephosphorylation of undecaprenyl pyrophosphate (C55-PP) to undecaprenyl phosphate (C55-P), which prevents the formation of lipid I/II and the eventual disruption of the peptidoglycan layer [17]. Additionally, recent scanning-electron microscopy (SEM) analysis has shown that bacitracin inhibits the formation of biofilm by *Streptococcus mutans* by downregulating several genes related to cell division and biofilm [16].

Glucosamine-6-phosphate synthetase (G6PS) is an enzyme that catalyzes the production of uridine diphosphate N-acetylglucosamine (UDP-GlcNAc), which is a precursor for peptidoglycan synthesis [18]. Bacilysin (2) and its chlorinated derivative chlorotetaine (3) (Figure 3) were first isolated from *B. subtilis* A14 and *B. amyloliquefaciens* ZJU-2011, respectively [19,20]. Both compounds are active against a broad range of bacteria, with bacilysin inhibiting *E. coli* at MIC = 0.001 µg/mL and chlorotetaine inhibiting *Candidas* spp. and *Aspergillus niger* at an MIC value of 1.8–7.8 μg/mL [20,21]. Bacilysin first enters the cell by binding to a transmembrane transport protein and is subsequently hydrolyzed to anticapsin, a G6PS inhibitor [22]. Kanosamine (4) (Figure 3) produced from *B. cereus* UW85 inhibits a wide array of plant-related pathogens (i.e., *Phytophthora medicaginis* M2913 with an MIC = 25 µg/mL) [23]. Kanosmine inhibits *Candida albicans* by utilizing the glucose transport system to transport itself into the cell, where it is subsequently phosphorylated to kanosamine-6-phosphate [24]. Kanosmine-6-phosphate inhibits G6PS, leading to the in septum deformation and cell agglutination of *C. albicans*.

**Figure 4:**
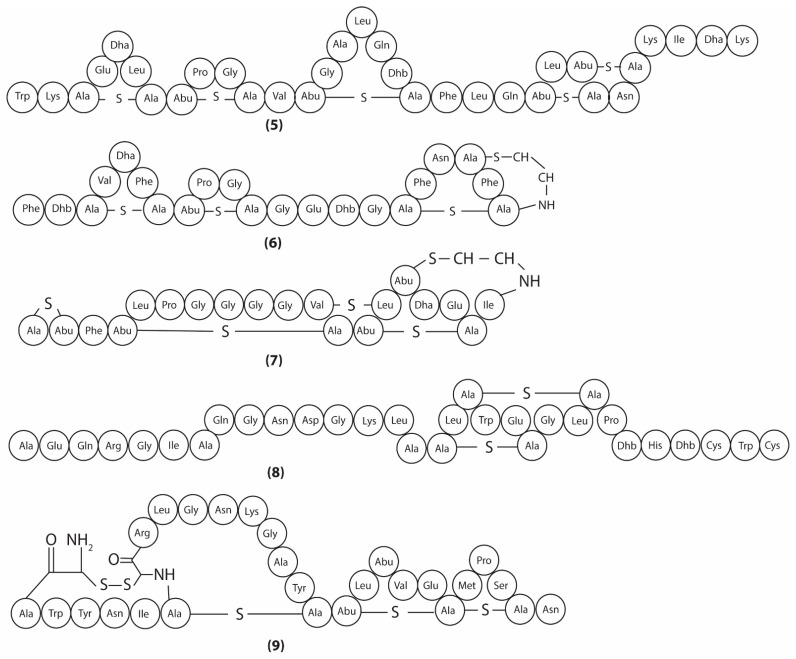
Chemical structures of subtilin (**5**), clausin (**6**), mersacidin (**7**), amylolysin A (**8**), and haloduracin (**9**).

Lipid II is a peptidoglycan intermediate, which is formed when the glycosyltransferase MurG catalyzes the addition of N-acetylglucosamine (GlcNAc) to lipid I [25]. Lipid II subsequently translocates across the plasma membrane, where it transfers MurNaC and GlcNAc to the peptidoglycan layer [26] (Figure 2a). Lipid II is generally conserved throughout microbes and has been studied as a target for various antimicrobial compounds, especially lantibiotics [27]. Lantibiotics are a class of large ribosomal compounds, typically around 3000kDa, and contain unique lanthionine and β-methyllanthionine residues [28]. These lantibiotics are often further divided based on the enzymes involved in their biosynthesis, which includes class I (5, 6) and class II (7, 8, 9) lantibiotics (Figure 4). Subtilin (5) is a class I lantibiotic isolated from *B. subtilis* 6633 [29]. This metabolite inhibits gram-positive bacteria, with MIC of 0.05 µg/mL (*Micrococcus luteus* NDCO8166) [29]. Binding studies show that subtilin binds to lipid II and pyrophosphate-containing intermediates. These pyrophosphate intermediates coat the outer cell membranes, and subtilin attaches to these intermediates, forming membrane pores [30]. These pores release essential metabolites, which eventually lead to cell death. Clausin (6), a class I lantibiotic produced by *B. clausii* O/C, inhibits gram-positive microbes (e.g., *Micrococcus luteus*, *MRSA* with MICs = 16 mg/L and 128 mg/L respectively) [31,32]. Clausin interacts with both lipid I/II and GlcNAc, forming stable complexes, which obstruct its role in peptidoglycan biosynthesis and hindering microbial growth [31].

A class II lantibiotic, mersacidin (7), was first isolated from *Bacillus* sp. HIL Y-85,54728 and shows activity against a range of gram-positive bacteria including *Staphylococcus aureus* SG511 with an MIC = 1 μg/mL [33,34]. Mersacidin associates with lipid II, which interferes with peptidoglycan biosynthesis and obstructs the growth of the microbe [35]. The class II lantibiotic amylolysin A (8), produced by *B. amyloliquefaciens* GA1, targets gram-positive bacteria such as *Enterococcus faecium* RFB128 with a MIC = 0.3µg/mL [36]. Amylolysin A exerts its antimicrobial effect by two separate mechanisms [37]. First, amylolysin A interacts with lipid II to hinder the biosynthesis of peptidoglycan. Secondly, amylolysin A induces the formation of membrane pores, leading to cell lysis. Haloduracin (9), a class II lantibiotic isolated from *B. halodurans* C-125, targets gram-positive bacteria such as *Lactococcus lactis* HP ATCC 11602 (MIC = 0.4 μg/mL) [38]. Structural analysis has highlighted that haloduracin is comprised of two parts, Halα and Halβ. Halα binds to lipid II in a 2:1 stoichiometry, preventing peptidoglycan biosynthesis. Halβ (2330 Da), however, binds to the anionic lipids of the cell membrane, resulting in pore formation [39].

### 3.2. Metabolites Targeting Plasma Membrane

The plasma membrane is composed of a phospholipid bilayer, which separates the intracellular compartment from the extracellular environment and may selectively transport metabolites across the membrane [40]. From the reviewed 47 *Bacillus* metabolites, 23 were identified to target different processes of the cell membrane (Figure 2b).

**Figure 5:**
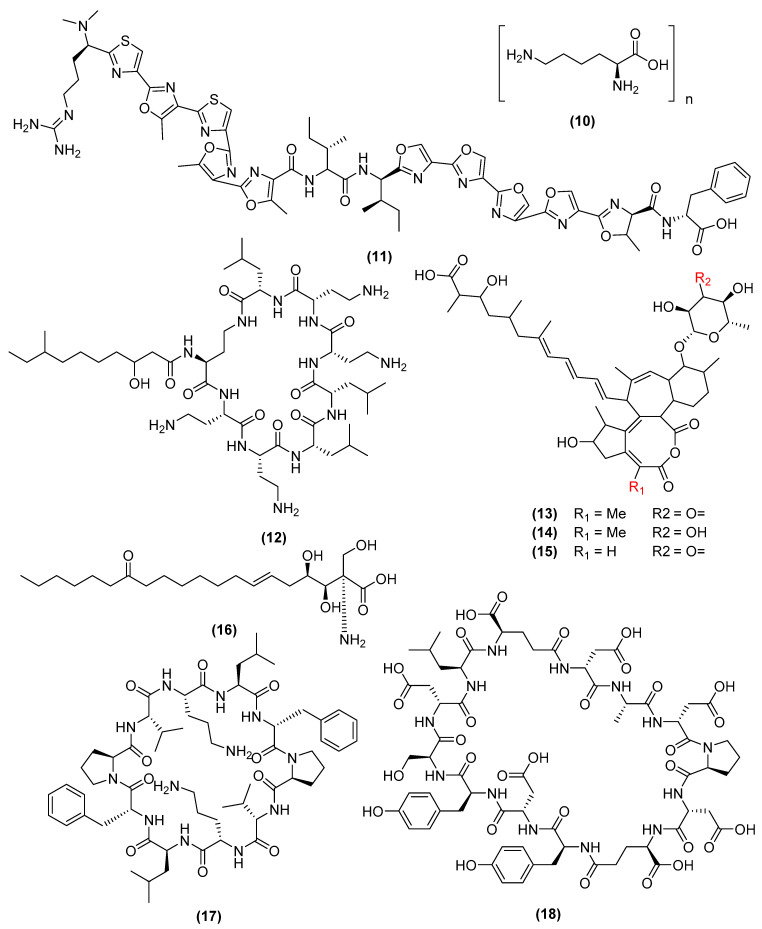
Chemical structures of ε-poly-L-Lysine (**10**), plantazolicin (**11**), octapeptin B (**12**), aurantinin B (**13**), aurantinin C (**14**), aurantinin (D) (**15**), myriocin (**16**), gramicidin A (**17**), and gramicidin S (**18**).

The lipid bilayer controls the permeability and shape of the plasma membrane and is affected by the negative-charged outer phospholipid layer [41]. Any changes to this membrane, whether by altering its lipid composition or the phospholipid layer, may distort its function as a barrier to the extracellular environment, releasing essential ions from the cell, eventually leading to cell death. ε-Poly-L-lysine (10) (Figure 5) is a homopolymer produced from *B. subtilis* SDNS, which exerts antimicrobial activity against gram-positive and gram-negative bacteria, as well as fungi (e.g., 600 μg/mL for *Ralstonia solanacearum*) [42]. ε-Poly-L-lysine electrostatically attaches to the phospholipid layer of the plasma membrane, which disturbs the membrane permeability to eventually lead to cell death [43,44]. Plantazolicin (11) (Figure 5), a product of *B. velezensis* FZB42, has been identified as a bacteriocin of interest, due to its restrictive spectrum against clinically relevant pathogens, such as *B. anthracis,* with an MIC value of 1–16 μg/mL [45]. This is highly relevant due to the very serious nature of anthrax. Further mechanism studies revealed that plantazolicin induces higher membrane fluidity and increases the proportion of cardiolipin, a cholesterol associated with higher osmotic stress [45]. Octapeptins are a class of lipooctapeptide antibiotics that were first isolated from *B. circulans* and that primarily inhibit gram-negative bacteria, with weaker activity on gram-positive bacteria and fungi [46]. Membrane microscopy studies show that octapeptin B (12) (*E. coli* SC 9251 MIC = 0.3 µg/mL) (Figure 5), produced from *B. circulans* ATCC 21656, disrupts the ion permeability of the membrane, which reduces the membrane proton gradient [47]. This translates into extensive membrane damage, the efflux of charged metabolites, and cell lysis. The aurantinins B-D (13–15) (Figure 5), a class of metabolites isolated from *B. subtilis* FMB60, exhibit similar MIC value for certain clinically relevant strains (i.e., *Clostridium sporogenes* CICC 10385 with a MIC ≤ 0.78 µg/mL, methicillin-resistant *Staphylococcus aureus* (MRSA) with an MIC = 6.25 µg/mL) [48]. SEM and transmission electron microscopy (TEM) studies show that the aurantinins cause plasma membrane lysis, leading to the efflux of metabolites from the cytoplasm [48]. However, these compounds require further structural elucidation to determine their precise stereochemistry. Myriocin (16) (Figure 5), produced from *B. amyloliquefaciens* LZN01, exerts antifungal activities against *Candidas albicans* (MIC = 1.0 µg/mL) [49]. SEM and TEM microscopy studies have indicated that myriocin binds to serine palmitoyl transferase and disrupts the plasma membrane, causing leakage and eventual pore formation [50]. Further omics analysis has revealed that myriocin alters the expression changes related to sphingolipid metabolism, glycerophospholipid metabolism, steroid biosynthesis, ABC transporters, and protein processing [51]. These genes are all relevant to the plasma membrane, suggesting that myriocin may target the expression of DNA. Gramcidins are a class of antibiotic decapeptides synthesized by *Aneurinibacillus migulanus* (formerly *B. brevis*) and consist of linear gramicidin A, B, C, and the circular gramicidin S. Gramicidin A (17) (Figure 5), a 15 amino-acid peptide, destroys gram-positive bacteria (*Streptococcus pyogenes* with a MIC = 33 nM) [52]. Unlike other antimicrobial metabolites, gramicidin A forms a single ion channel, which distorts the membrane and allows the passage of cations across the membrane [53]. Once inside, gramicidin A can also induce the formation of reactive oxygen species (ROS), which damages the intracellular DNA, mitochondria and triggers necrosis [54]. The gram-positive bacteria *Aneurinibacillus migulanus* (formerly *B. brevis natto*) inhibits several gram-positive, gram-negative, and fungi microbials (e.g., *Staphylococcus aureus* with a MIC value of 3.9 µg/mL) by producing gramicidin S (18) (Figure 5) [55]. Gramicidin S interacts with the plasma membrane by forming oligomeric β-barrel pores, which destroys the barrier properties of the membrane [56,57]. Further in vivo studies have shown that gramicidin S binds to the DNA and inhibits transcription and cell growth [58].

**Figure 6:**
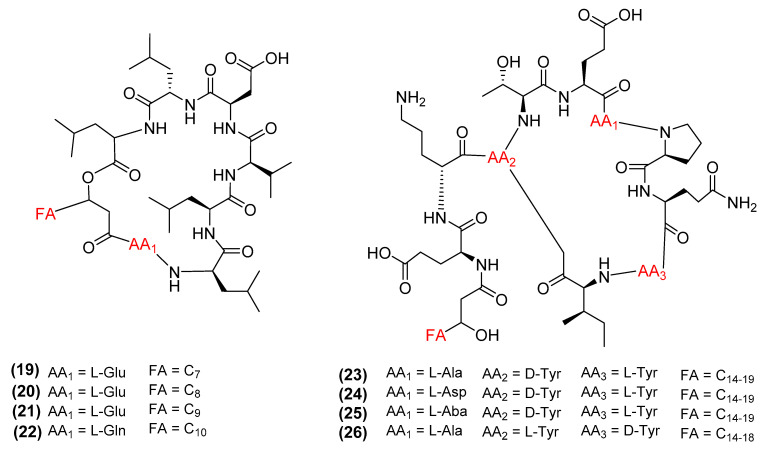
Chemical structures of surfactin A–C (**19**–**21**), lichenysin (**22**), and fengycin A–D (**23**–**26**).

Pore-formation metabolites act in a concentration-dependent manner, by forming ion-like channels that release vital ions from the cell, leading to cell death. At low concentrations, these metabolites form unilamellar vesicles on the outer lipid membrane, distorting the shape of the cell, and eventually, lead to apoptosis [59,60,61]. At higher concentrations, these metabolites aggregate to form pores at the plasma membrane, causing the leakage of nucleic acids, essential ions, and ATP from the cell to cause necrosis [59,62,63,64]. *Bacillus* metabolites that typically utilize this mechanism includes the class of compounds known as lipopeptides. Lipopeptides are composed of a cyclic oligopeptide, attached to a flexible lipid tail, and consist of several groups including the surfactins, fengycins, and iturins [65]. Surfactins were first isolated from a culture broth of *B. subtilis* and include the compounds surfactin A (19), B (20), C (21), and lichenysin (22) (Figure 6) [66]. Surfactins exert their antibacterial activities by acting on the plasma membrane through the pore-forming mechanism [67]. Additionally, surfactins (21-22) can breakdown bacterial biofilms by decreasing the percentage of alkali-soluble polysaccharides and downregulating the expression of genes involved in biofilm formation such as icaA and icaD [68]. Lastly, surfactins can also induce the grapevine immune system in response to infection [69]. Fengycins (23-26) (Figure 6) are antifungal lipopeptides first isolated from *B. subtilis* F-29-3 (e.g., *Rolani stolonifera* with a MIC = 400 µg/mL) [70]. These fengycin molecules are often reported as membrane disruptors, either by deforming membrane shape or by causing pores, leading to cell death [71]. More recent studies have additional antimicrobial mechanisms of action for fengycin A (23) and fengycin B (24). Fengycin A can alter the gene expression related to cell wall synthesis, which alters cell components and increases hydrophobicity [72]. Furthermore, fengycin B155, a mixture of fengycin A (23) and fengycin B (24), is able to disrupt multiple intracellular components of the cell [73]. These processes include the inhibition of the mitochondria membrane potential, the condensation of chromatin involved in replication, the cleavage of DNA repair protein (poly (ARP-ribose) polymerase), and the accumulation of ROS [73]. Lastly, fengycins have been shown to inhibit quorum sensing, due to their structural similarity to *S. aureus* accessory gene regulator (Agr) [74]. Agr is a virulence factor that mediates the cell-to-cell communication between cells, and its inhibition prevents the aggregation and biofilm formation needed to promote survival [75]. Plipastatin A (26) is a lipopeptide commonly associated with the fengycin family due to its structural similarity and antifungal properties (*Fusarium oxysporum* with a MIC = 16 μg/mL) [76]. TEM analysis demonstrated that plipastatins disrupt the cell wall, membrane, and cytoskeleton of *Fusarium oxysporum*, causing intracellular leakage and eventual cell death.

**Figure 7:**
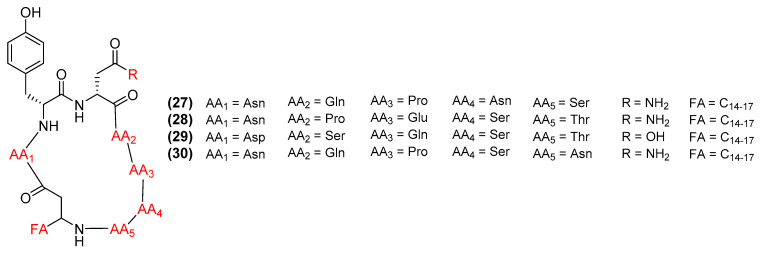
Chemical structures of iturin A (**27**), bacillomycin D (**28**), bacillomycin L (**29**), and mycosubtilin (**30**).

Iturins (27–30) (Figure 7) are cyclic lipopeptides that includes iturin A (27), bacillomycin D (28), bacillomycin L (29), and mycosubtilin (30) [77]. These peptides primarily inhibit fungi by binding to the cell membrane with its fatty acid tail to form ion-conducting or phospholipid–lipopeptide sterol complexes [78]. Optical and fluorescence microscopy studies have revealed that iturin A (27) severely damages the plasma membranes of *Fusarium graminearum* at a MIC = 5 µg/mL by forming a large pore and inhibiting hyphae growth [79]. Iturin A can stimulate oxidative stress, leading to mitochondria damage and the eventual destruction of the cell [80]. Lastly, iturin A increases the transcription of immune defense genes in several plants [81]. Bacillomycin D (28) exerts antifungal properties against *Colletotrichum gloeosporioides* with an MIC of 2.2 µg/mL [82,83]. SEM and TEM analysis confirmed bacillomycin D’s ability to target both cell wall and plasma membrane, leading to the leakage of intracellular organelles [82]. Bacillomycin D can disrupt the cell membrane by upregulating the expression of genes involved in ergosterol synthesis and oxidative stress [84]. These sterols adjoin to the membrane, distorting its shape and eventually releasing vital intracellular components to the environment [84]. Additionally, bacillomycin D can increase the expression of specific genes to produce ROS molecules and cellular antioxidant enzymes including deoxyivalentol, glutathione reductase, and thioredoxin [85]. Bacillomycin D has also been reported to act as a biofilm activator by binding to the matrix complex KinB-Spo0A-SinI-SinR, which triggers the production of biofilm [86]. Lastly, bacillomycin D stimulates the expression of genes involved in mediated defense responses and enzymatic proteins that can be released to target competing growth [86]. *B. amyloliquefaciens* K103 produces the potent antifungal metabolite bacillomycin L (29) (*Saccaromyces cerevisiae* with a MIC = 30 µg/mL) [78,87]. Like other iturins, bacillomycin L primarily acts on the plasma membrane, forming pores that releases its intracellular components outside the cell [88]. Studies have shown that bacillomycin L binds to sterols on the membrane, destroying the membrane and killing the cell [89]. Bacillomycin L can also alter the expression of 39 different genes in *Rhizoctonia solani* related to cellular stress, such as calcium homeostasis, energy metabolism, protein degradation, RNA processing, and carbohydrate metabolism [90]. Mycosubtilin (30), an antibiotic from the iturin group, inhibits the growth of fungal *Saccharomyces cerevisiae* with a MIC of 10 µg/mL [78]. Increased concentrations of mycosubtilin causes the lysis of the phospholipid layer, either by the aggregation of lipopeptides or clustering of mycosubtilin [91]. This binding increases membrane permeability, leading to metabolite release and the eventual lysis of the cell [92]. Mycosubtilin can also activate the salicylic acid and jasmonic acid signaling pathways involved in the immune response to pathogenic microbes [69].

**Figure 8:**
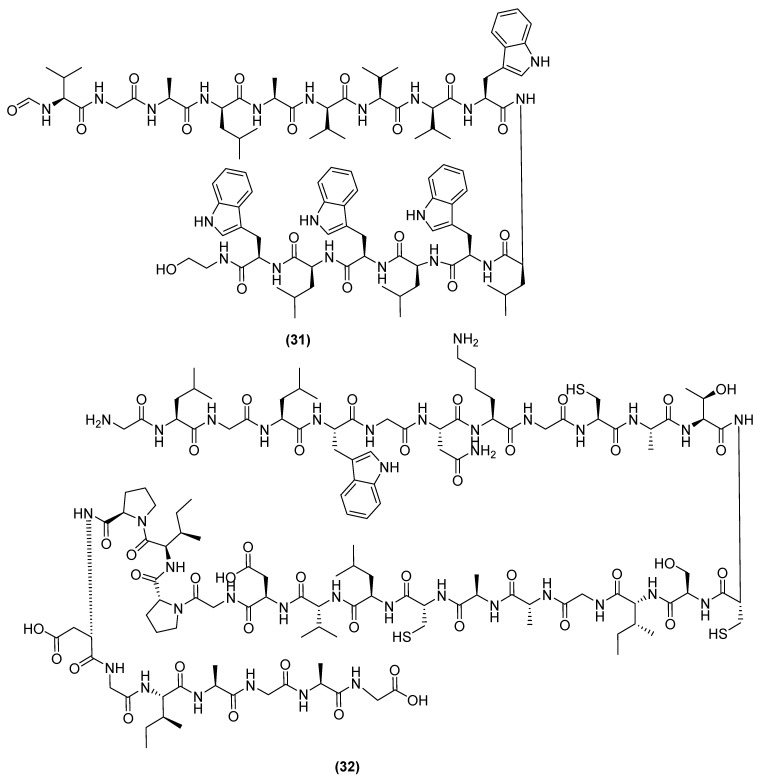
Chemical structures of mycobacillin (**31**) and subtilosin A (**32**).

Mycobacillin (31) (Figure 8), an antifungal polypeptide sourced from *B. subtilis* B3, is active against *Aspergillus niger* at 20 µg/mL [93,94]. Mycobacillin has been reported to bind to ATP transporter on the plasma membrane, leading to the excessive release of ATP and the subsequent starvation of the cells [94,95]. Subtilosin A (32) (Figure 8) is a sactipeptide produced by *B. subtilis* 168 that processes antibacterial activity against both gram-positive and gram-negative pathogens (i.e., *Gardnerella vaginalis* MIC = 7.2 µg/mL) [96,97]. Its specific mechanism of action involves subtilosin A anchoring to a membrane receptor, whilst electrostatically binding to the plasma membrane [98]. This electrostatic binding dissipates the transmembrane pH gradient, causing an efflux of intracellular ATP that starves the cell and eventually leads to its death. Subtilosin A has also been shown to inhibit biofilm formation, presumably by blocking quorum sensing between cells [99].

### 3.3. Metabolites Targeting Intracellular Processes

*Bacillus* metabolites may cross the plasma membrane and bind to several intracellular targets essential for cell survival. These intracellular processes include DNA transcription, RNA translation, and protein metabolism needed for energy production. Transcription is the first step in gene expression, in which information from a gene is used to construct a functional product such as a protein. For a protein-coding gene, the RNA copy, or transcript, carries the information needed to build a protein. From the 47 compounds reviewed in this paper, 11 compounds primarily target the intracellular processes.

**Figure 9:**
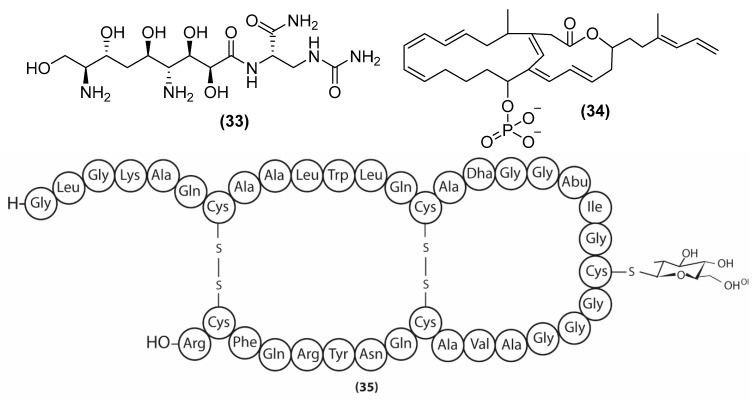
Chemical structures of zwittermicin A (**33**), difficidin (**34**), and sublancin (**35**).

Zwittermicin A (33) (Figure 9), an aminopolyl antibiotic produced by *B. cereus* UW85, inhibits gram-positive and gram-negative bacteria, as well as fungi (i.e., *Erwinia herbicola* L S005 with a MIC = 60 µg/mL) [100]. Zwittermicin A disrupts cellular growth by targeting either DNA transcription and replication via inhibition of two enzymes, gyrase and topoisomerase [101]. Difficidin (34) (Figure 9), a highly unsaturated macrolide phosphate first isolated from *B. subtilis* ATCC 39320, can inhibit both gram-positive and negative strains such as *Rolani solanacearum* with a MIC value of 12.62 µg/mL of [102,103]. Microscopy analysis has revealed that difficidin downregulates the genes related to cell wall synthesis, protein production, and DNA replication [104]. Sublancin (35) (Figure 9), a glycosylated peptide produced by *B. subtilis* 168, displays antibacterial activities (i.e., methicillin-resistant *Staphylococcus aureus* ATCC43300 with a MIC = 15 μM) [105]. Mechanism investigations suggest that sublancin enters the cytoplasm and reduces DNA transcription and translation [106].

**Figure 10:**
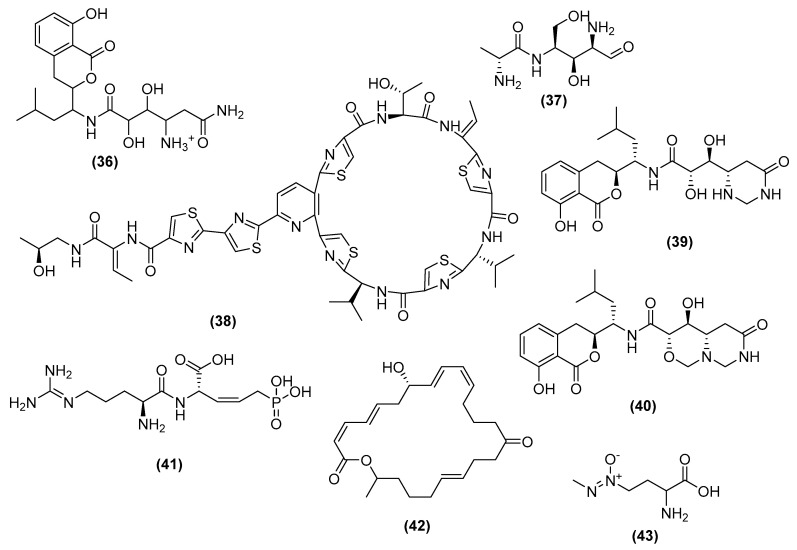
Chemical structures of amicoumacin A (**36**), prumycin (**37**), thiocillin (**38**), hetiamacin E (**39**), hetiamacin F (**40**), rhizocticin A (**41**), macrolactin N (**42**), and azoxybacilin (**43**).

The amicoumacins are a class of dihydroisocoumarin compounds, produced by *B. pumilus,* that exert antibacterial, antifungal, and anti-inflammatory properties. In particular, amicoumacin A (36) (Figure 10), produced by *B. pumilus* BN-103, inhibits *B. subtilis* 1779 with an MIC = 20.0 µg/mL. Further studies have shown that amicoumacin A inhibits the protein synthesis of methicillin-resistant *Staphylococcus aureus* by stabilizing the mRNA at the terminal E site on the ribosome during protein synthesis [107]. This disruption results in the perturbation of the membrane, leading to energy dissipation and eventual cell death [107,108]. Prumycin (37) (Figure 10), isolated from a culture broth of *B. amyloliquefaciens* SD-32, exerts bactericidal and fungicidal effects, such as on *S. sclerotiorum,* with an MIC value of 1.56 µg/mL [109,110,111]. Prumycin inhibits the protein synthesis of *Sacrina lutea*, preventing the activation of amino acids needed for protein synthesis and the transfer of amino acids to RNA [110].

Thiocillin (38) (Figure 10), produced by *B. cereus* ATCC 14579, has been previously reported to only target gram-positive bacteria but has recently been shown to also target gram-negative bacteria [112]. Its mechanism on gram-positive bacteria works by targeting the 50S ribosome and inhibiting its role in protein synthesis [113]. In contrast, thiocillin targets the gram-negative bacterium *Pseudomonas aeruginosa* by binding to ferrioxamine receptor FoxA, which disrupts the proton motive force to inhibit translation [113]. Hetiamacin E and F (39–40) (Figure 10) produced from *B. subtilis* PJS display antibacterial activity against methicillin-resistant *Staphylococcus aureus,* with MIC values of 8–16 µg/mL and 32 µg/mL, respectively [114]. Hetiamacin E and F inhibit protein biosynthesis, resulting in the disruption of mRNA translation, leading to cell death [114]. Rhizocticin A (41) (Figure 10) is a potent antifungal first produced from *B. subtilis* 6633. Its bioactivity data shows that it is active against a range of budding and filamentous fungi (bioactivity not avaliable) [115]. Mutant analysis suggests that rhizocticin utilizes the peptide transport system to enter the cytoplasm, where it forms the fungitoxic L-2-amino-5-phosphono-3-cis-pentenioc acid (L-APPA). L-APPA interferes with threonine metabolism, which inhibits cell growth [116].

Macrolactin N (42) (Figure 10), a novel macrolactin produced by *B. subtilis* A29, is shown to inhibit *Staphylococcus aureus* peptide deformylase (PDF), with an MIC of 100 µM [117]. PDFs are essential bacterial specific metalloenzymes, which removes formyl groups during polypeptide elongation [117]. The inhibition of these PDFs leave bacteria unable to hydrolyze these polypeptides and hinder its ability to synthesize proteins [117]. Azoxybacilin (43) (Figure 10), first isolated from *B. cereus* NR2991 and *B. cereus* Frankland, is active against a broad spectrum of mycelial fungi, such as *Candida albicans* (IC_50_ = 1.2 mg/mL) [118,119]. Its mechanism involves the interruption of the sulfur fixation pathway, an essential support system for microbial growth, by decreasing the expression of sulfate assimilation genes including MET10 and MET4 [118]. MET10 regulates the expression of sulfite reductase, and MET4 is the transactivator of MET10. The reduction of the gene expression in the sulfur-fixation pathway disrupts this support system and eventually leads to cell growth inhibition.

### 3.4. Metabolites Interacting with Other Emerging Targets

**Figure 11:**
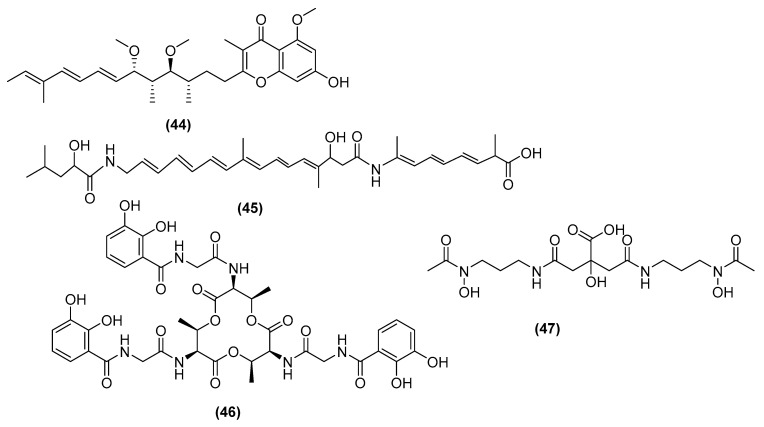
Chemical structures of stigmatellin Y (**44**), bacillaene (**45**), bacillibactin (**46**), and schizokinen (**47**).

Quorum sensing, also known as cell-to-cell communication, is the regulation of a microbial gene expression in response to its cell density [120]. This mechanism relies on small chemical indicators and has been linked to pathogen virulence, due to its effect on cell reproduction, mobility, and biofilm formation [121]. Biofilms are extracellular adhesive structures produced by various strains of bacteria that assist in their tolerance to UV, acidity conditions, and vulnerability to antimicrobial metabolites [122]. Several key groups of *Bacillus* metabolites have been shown to interfere with this process [123]. Nonetheless, *Bacillus* metabolites such as stigmatellin Y (44) (Figure 11) have been identified as a biofilm inhibitor [124]. Stigmatellin Y is shown to inhibit *Pseudomonas aeruginosa* biofilm formation, presumably by acting as a competitive inhibitor to the quorum sensing mediator PqsR [124]. Bacillaene (45) (Figure 11) has been identified as a biofilm inhibitor produced by numerous *B. subtilis* strains [125]. Analysis of mutant strains revealed that bacillaene inhibits the biofilm of *Campylobacter jejuni*, preventing the formation of microcolonies and eventually disrupting their microbial growth.

Siderophores are small molecules secreted by microorganisms that are involved in iron (Fe^2+^) uptake from the environment [126]. Iron is an essential metabolite for microbial growth and strategies have been developed to starve pathogenic microorganisms using these siderophores. Siderophores produced by *Bacillus* strains include bacillibactin (46) and schizokinen (47) (Figure 11), which were first isolated from *B. subtilis* and *B. megaterium* ATCC 19213, respectively [127,128]. These metabolites facilitate the uptake of ferric ions (Fe^3+^) from the environment to the bacterial cell using specific membrane receptors to enter the host cell [129]. Once inside, these ions are reduced to ferrous (Fe^2+^) ions for use in microbial growth [130].

## 4. Conclusions Remarks and Future Directions

**Figure 12:**
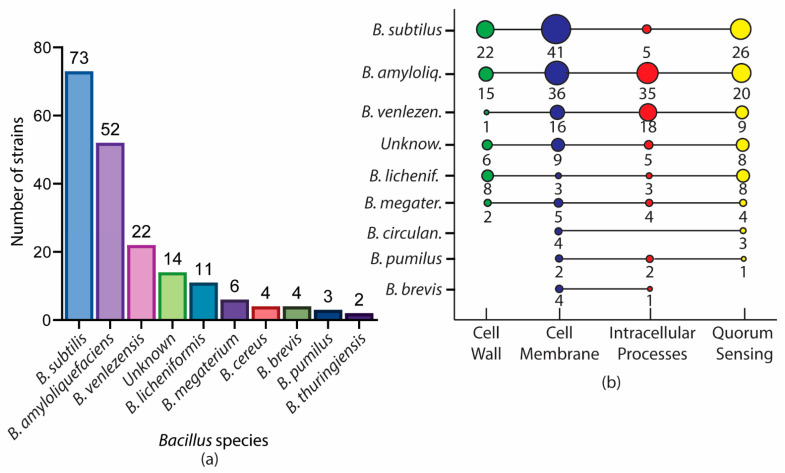
The analysis of (**a**) the number of strains in each species and the (**b**) mechanism of action targeted by each strain.

This paper reviews the current literature on antimicrobial compounds from *Bacillus* sp. and their mechanism of action. Further analysis on the source of antimicrobial compounds and their mechanism of action revealed some interesting trends. In terms of number of strains that produce antimicrobial metabolites, the most prolific is *subtilis* (*n* = 73), followed by *amyloliquefaciens* (*n* = 52) and *velezensis* (*n* = 22) (Figure 12a). *B. subtilis* is a common bacterium in soil and one of the most-studied *Bacillus* sp. Research has shown that these species are strongly related to each other, with several papers suggesting that *amyloliquefaciens* be renamed as *velezensis* due to its similarity in conserved genomic sequence [131,132]. The least reported of these *Bacillus* sp. is *B. thuringiensis*, with only two strains producing antimicrobial compounds in the literature. This highlights the lack of studies for this species and may warrant further investigation.

Further analysis on mechanism of action (Figure 12b) reveals that the cell membrane is the most popular target of different species of *Bacillus* and their metabolites (*n* = 122), followed by quorum sensing (*n* = 79), intracellular processes (*n* = 73), and the cell wall (*n* = 57). Quorum sensing is an interesting emerging target, as more species and metabolites (*n* = 79) hinder the process and hence, inhibit cell-to-cell communication. Further analysis also notes that many *Bacillus* species and their metabolites exert their antimicrobial activity through not only one but multiple mechanisms.

Several publications noted the geographic location of *Bacillus,* as well as the source of the bacteria. Further analysis based the information provided in the literature reveals that the majority of identified strains are from Asia (*n* = 37), followed by South America (*n* = 8) and the Middle East (*n* = 4). This observation may indicate that these strains share genomic similarities or properties, however, it may also stem from the research laboratories located in these sites and could be a byproduct of a focus on probiotic research at these locations. Additionally, the top three sources that these strains were isolated are from soil, local produce, and waterways. These findings reinforce the use of soil-based screening as a rich source of microorganisms. It also highlights the recent trend in investigating food produce as a source of *Bacillus* isolates. This is either guided by historical evidence of their antimicrobial properties or the anecdotal knowledge of their safe use and consumption.

The advancements of omics technologies are essential for the rapid screening of future probiotics. The characterization of the genome and biochemical properties allows the selection of particular strains with properties suitable for industrial use. A number of omics techniques have been developed to provide valuable information on the characteristics, optimization, and metabolic pathways behind antimicrobial activity [133]. One example uses omics to a rapid screen of selected *Bacillus* strains for specific gene markers known for antimicrobial activity [133]. For example, the genomic screening of *B. velezensis* CC09 revealed the loci for iturin A previously not identified in its initial screening [134].

In-depth analysis of these pathways and the precursors may reveal optimal conditions needed to produce these metabolites [135]. Wiegand utilized metabolomics and genome mining to provide insight into the expression of DNA under various fermentation conditions. These conditions includes pH levels, temperatures, and oxygen levels, which result in the discovery of optimal conditions needed to express the antimicrobial gene of interest and maximizing their yield [136]. This technique, alongside computational modelling systems, may reveal other conditions unexplored such as the ratio of carbon to nitrogen in fermentation media and the presence of small metabolites and co-culturing in order to further maximize the production of antimicrobial metabolites. As production is required, especially when optimizing for commercial purposes, these techniques can open up the field in the use of bacteria as a source of antimicrobial compounds to tackle the declining rate of antimicrobial compounds being discovered.

## Supplementary Materials

The following are available online at https://www.mdpi.com/article/10.3390/antibiotics11010088/s1, Table S1: 47 antimicrobial metabolites from *Bacillus*. References [137,138,139,140,141,142,143,144,145,146,147,148,149,150,151,152,153,154,155,156,157,158,159,160,161,162,163,164,165,166,167,168,169,170,171,172,173,174,175,176,177,178,179,180,181,182,183,184,185,186,187,188,189,190,191,192,193,194,195,196,197,198,199,200,201,202,203,204,205,206,207,208,209,210,211,212,213,214,215,216,217,218,219,220,221,222,223,224,225,226,227,228,229,230,231,232,233,234,235,236,237,238,239,240,241,242,243,244,245,246,247,248,249,250,251] are cited in the supplementary materials.
