# Supplementary material for: Antimicrobial Bacillus: Metabolites and Their Mode of Action
Source: Antibiotics (Basel). 2022 Jan 12;11(1):88. doi: 10.3390/antibiotics11010088 (PMC8772736; doi:10.3390/antibiotics11010088)
Supplement: Supplementary file 1 [file antibiotics-11-00088-s001.zip › antibiotics-1530899-supplementary.pdf]

223Antimicrobial *Bacillus*: metabolites and their mode of action

charlie tran, Ian E. Cock, Xiaojing Chen and Yunjinag Feng

Table S1. 47 antimicrobial metabolites from *Bacillus*.

Metabolite	Source strain	Molecular target	Indicator strain	Bioactivity (MIC or other-wise indicated)	Source location	Reference
Bacitracin A	N/A	Cell wall/Biofilm	Staphylococcus mutans MTCC 497	78.12 µg/ml	N/A	[16]
	B. GU057	Cell wall/Biofilm	Staphylococcus aureus<br>Micrococcus luteus	ZoI = 18mm<br>ZoI = 13mm	Saline soil in the roots of Terminalia Arjuna roots in District D.I.Khan at pH 8.2.	[137]
	B. licheniformis DW2	Cell wall/Biofilm	N/A	N/A	N/A	[138]
	B. licheniformis 10716	Cell wall/Biofilm	N/A	N/A	N/A	[139]
	B. licheniformis strain AL	Cell wall/Biofilm	N/A	N/A	N/A	[139]
	B. licheniformis 16	Cell wall/Biofilm	N/A	N/A	N/A	[140]
	B. licheniformis 28 KA	Cell wall/Biofilm	Bacillus licheniformis	N/A	N/A	[141]
	B. subtilis C 126	Cell wall/Biofilm	Micrococcus flavus N/A	N/A	Sugar Cane	[142]
	B. licheniformis A-5	Cell wall/Biofilm	N/A	N/A	Chinese liquor-making process	[142]
	B. subtilis MH-4	Cell wall/Biofilm	N/A	N/A	N/A	[143]
	B. spp. GU215	Cell wall/Biofilm	Staphylococcus aureus	ZoI = 18mm	N/A	[144]
	B. paralicheniformis UBBLi30	Cell wall/Biofilm	Micrococcus luteus<br>MRSA<br>Streptococcus aureus<br>Streptococcus Pyrogenes<br>Propionibacterium Acnes<br>Micrococcus luteus MTCC 106<br>MRSA	ZoI = 30.6mm<br>ZoI = 9.6mm<br>ZoI = 10.6mm<br>ZoI = 10.3mm<br>ZoI = 11.0mm<br>0.015625 mg/l<br>8 mg/l	Traditional fermented food and preserved in culture collection of Unique Biotech Limited, India	[32]
Bacilysin			E. coli	0.001 µg/ml		[21]
	B. amyloliquefaciens X030/ (KM191359.1)	Cell wall	N/A	N/A	Peanut soil in Henan province	[145]
	B. subtilis A14	Cell wall	N/A	N/A	N/A	[146]
	B. subtilis 168	Cell wall	N/A	N/A	N/A	[147]
	B. subtilis ME488	Cell wall	N/A	N/A	Korean greenhouse soils	[148]
	B. sp. strain CS93	Cell wall	N/A	N/A	Pozol, Villahermosa, Tabasco, Mexico	[149]
	B. amyloliquefaciens ZJU-2011	Cell wall	N/A	N/A	East China Sea	[20]
	B. amyloliquefaciens MTCC 10456	Cell wall	Malassezia furfur ATCC 44344,<br>Malassezia furfur ATCC 12078,<br>Malassezia globose ATCC MYA 4612	50-100 µg/mL<br>50-110 µg/mL<br>30-100 µg/mL	Seaweed	[150]
	B. luciferensis K2	Cell wall	N/A	N/A	Organic soils of Sikkim, India	[151]
	B. amyloliquefaciens K12	Cell wall	N/A	N/A	Organic soils of Sikkim, India	[151]
	B. subtilis BioCWB	Cell wall	N/A	N/A	Organic soils of Sikkim, India	[151]

Kanosamine	Bacillus cereus UW85	Cell wall	Aphanomyces euteiches WI-98,<br>Pythium torulosum A25A,<br>Pythium aphanidermatum Pa138<br>Phytophthora medicaginis M2913<br>Aspergillus flavus MP03<br>Botrytis cinerea NRRL1684<br>Candida utilus 1Y0-Y002<br>Ophinostoma ulmi UWCC82<br>Saccharomyces cerevisiae Y008<br>Sclerotinia homoeocarpa KS20<br>Sclerotinia sclerotiorum 91-26<br>Venturia inaequalis UWCC365<br>Rhizoctonia solani AG1, AG4<br>Tyhula incarnata SM93-34<br>Ustilago maydis 521<br>Alternaria alternata NRRL20593<br>Alternaria tagetica UWCC70<br>Colletotrichum phomoides UWCC37<br>Colletotrichum trifolii SMM<br>Cytospora cincta NRRL5185<br>Drechslera poae KS58<br>Epicoccum nigrum NRRLA-10128<br>Fusarium oxysporum UWCC62r1<br>Fusarium solani 93.21<br>Fusarium sporotrichioides CN-Z<br>Helminthosporum carbonum UWCC48<br>Helminthosporium sativum UWT84<br>Phomopsis obsurans UWCC95<br>Verticillium dahlia RNS87:1<br>Verticillium albo-strum Linden	40 µg/mL<br>150 µg/mL<br>250 µg/mL<br>25 µg/mL<br>Active	Root of a field-grown alfalfa plant from Arlington, WI	[23]
Chlorotetaine	B. amyloliquefaciens ZJU-2011	Cell wall	Candida albicans ATCC 10731<br>Candida albicans ATCC 10231<br>Candida parapsilosis JCM1785<br>Candida krusei CBS573<br>Candida parapolymorpha ATCC 26012<br>Aspergillus niger	7.8 µg/ml<br>3.9 µg/ml<br>3.9 µg/ml<br>1.8 µg/ml<br>1.8 µg/ml<br>3.9 µg/ml	East China Sea	[20]
	B sp. strain CS93	Cell wall	N/A	N/A	Mexican Fermented Maize Dough	[149]
Subtilin	B. amyloliquefaciens B-1895	Cell wall/ Cell membrane/ Biofilm	Lactococcus lactis MG 1614<br>Micrococcus luteus	1.1 µg/ml<br>0.05 µg/ml	Soil isolate (Russian National Collection of Industrial Microorganisms, Moscow, Russia)	[29,30,152]
	B. luciferensis K12	Cell wall/ Cell membrane/ Biofilm	N/A	N/A	Organic soils of Sikkim, India	[151]
	B. luciferensis strain K2	Cell wall/ Cell membrane/ Biofilm	N/A	N/A	Organic soils of Sikkim, India	[151]
	B. subtilis strain BiocWB	Cell wall/ Cell membrane/ Biofilm	N/A	N/A	Organic soils of Sikkim, India	[151]
Clausin	B. clausii O/C	Cell wall	Staphylococcus aureus CIP 350 53 156<br>Enterococcus faecium LMBA 27323<br>Micrococcus sp. LMBA 26<br>Lactococcus lactis ATCC 11454<br>Lactococcus lactic LMBA 514	Active<br>Active<br>Active<br>Active<br>Active	N//A	[31]

	B. clausii UBBC07 (MTC 5472)	Cell wall	Micrococcus luteus<br>Listeria monocytogenes<br>Enterococcus faecium<br>Enterococcus faecalis<br>Clostridioides difficile<br>Staphylococcus aureus<br>MRSA	20.5 mg/L<br>16.9 mg/L<br>11.9 mg/L<br>10.8 mg/L<br>12.7 mg/L<br>15.3 mg/L<br>13.7 mg/L	N/A	[153]
		Cell wall	Micrococcus luteus<br>MRSA	16 mg/L<br>128 mg/L		[153]
Mersacidin	N/A	Cell wall	Staphylococcus aureus SG511<br>Staphylococcus aureus SA137/93A<br>Staphylococcus aureus SA137/93G	1 µg/ml<br>35 µg/ml<br>30 µg/ml		[34]
	B. HIL Y-85,54728	Cell wall	Micrococcus luteus ATCC 4698 Staphylo-coccus simulans 22	0.1 µg/ml<br>11 µg/ml	Mulund (salt pan), Maharashtra, India	[154]
	N/A	Cell wall	Staphylococcus aureus SH1000<br>Staphylococcus aureus R33 MRSA<br>Staphylococcus epidermidis NCTC11047<br>Streptococcus pneumoniae BAA-255<br>Micrococcus luteus ATCC 4698<br>Enterococcus faecium ATCC 19579<br>Enterococcus faecalis ATCC 29212<br>Enterococcus faecium 7131121 VRE	32 µg/ml<br>32 µg/ml<br>16, 32 µg/ml<br>2 µg/ml<br>1, 2 µg/ml<br>32 µg/ml<br>64 µg/ml<br>64 µg/ml		[155]
	Bacillus amyloliquefaciens subsp. plantarum B9601-Y2	Cell wall	N/A	N/A	wheat rhizosphere	[156]
Amylolysin A	B. amyloliquefaciens GA1	Cell wall/ Cell mem-brane	Micrococcus luteus ATCC 9341<br>Staphylococcus epidermis ATCC 1228<br>Staphylococcus aureus ATCC 25923<br>Staphylococcus aureus ATCC 43300<br>Staphylococcus aureus RFB127<br>Enterococcus faecalis ATCC 29212<br>Enterococcus faecalis RFB129<br>Enterococcus faecium RFB128<br>Listeria monocytogenes LMG 23905<br>Listeria monocytogenes LMG 21263<br>Listeria monocytogenes LM2234<br>Listeria innocua ATCC33090<br>Listeria innocua RFB159<br>Listeria ivanovii RFB160<br>Bacillus cereus RFB125<br>Bacillus subtilis ATCC 6633<br>Bacillus megaterium RFB124<br>Streptococcus agalactiae RFB141<br>Weissella sp RFB139<br>Lactobacillus plantarum RFB138<br>Escherichia coli RFB149<br>Pseudomonas aeruginosa RFB148<br>Cryptococcus neoformans IHEM3969<br>Saccharomyces cerevisiae RFY100	0.7 µM<br>2.8 µM<br>2.8 µM<br>0.4 µM<br>1.4 µM<br>1.4 µM<br>0.7 µM<br>0.1 µM<br>0.4 µM<br>0.5 µM<br>0.4 µM<br>0.7 µM<br>0.7 µM<br>0.8 µM<br>0.2 µM<br>1.4 µM<br>0.4 µM<br>2.8 µM<br>2.8 µM<br>> 2.8 µM<br>> 2.8 µM<br>> 2.8 µM<br>> 2.8 µM<br>> 2.8 µM	Strawberry	[37]
	B. subtilis S499	Cell wall/ Cell mem-brane	N/A	N/A	Cultivated soil in the Iturie region (Democratic Republic of Congo; Delcambe, 1965)	[157]

Haloduracin	B. halodurans C-125	Cell wall/ Cell membrane	Lactococcus lactis HP<br>Lactococcus lactic 481<br>Lactococcus lactis 11454<br>Vancomycin resistant Enterococcus faecium<br>Bacillus anthracis Sterne 7702<br>Bacillus subtilis<br>Methicillin-resistant Staphylococcus aureus<br>Staphylococcus aureus<br>Staphylococcus epidermidis 15X<br>Micrococcus luteus<br>Streptococcus mutans	73.4 nM<br>195 nM<br>625 nM<br>781 nM<br>677 nM<br>469 nM<br>4690 nM<br>1560 nM<br>313 nM<br>1250 nM<br>2500 nM	N/A	[38]
ε -poly-L-Lysine	B. subtilis SDNS	Cell membrane	Escherichia coli<br>Staphylococcus aureus<br>Enterococcus faecalis<br>Pseudomonas aeruginosa<br>Vibrio sp. N2,<br>Vibrio fluvalis<br>Vibrio bulificus	Active<br>Active<br>Active<br>Active<br>Active<br>Active<br>Active	Sea water in Alexandria	[43]
	N/A	N/A	Gardnerella vaginalis	33 µg/mL		[97]
Plantazolicin	B. velezensis FZB42	Cell membrane	Bacillus brevis ATCC 8246<br>Bacillus subtilis 168<br>Bacillus cereus ATCC 14579<br>Bacillus licheniformis ATCC 9789<br>Micrococcus luteus<br>Bacillus subtilis CU1065<br>Bacillus subtilis HB0042<br>Bacillus sphaericus<br>Paenibacillus granivorans<br>Bacillus megaterium 7A1	Active<br>Active<br>Active<br>Active<br>Active<br>Active<br>Active<br>Active<br>Active<br>Active	N/A	[158]
		Cell membrane	Bacillus anthracis Sterne 7702<br>Bacillus anthracis Sterne 34F2 A0517.1<br>Bacillus cereus 2002013145<br>Bacillus cereus 2002013146<br>Bacillus cereus 2002013100<br>Bacillus cereus 2002013102<br>Bacillus cereus ATCC 4342<br>Bacillus cereus ATCC 7064<br>Bacillus cereus CDC 32805<br>Bacillus cereus G9241<br>Bacillus megaterium 899<br>Bacillus mycoides 96/3308	1<br>2<br>>64<br>>64<br>>64<br>>64<br>>64<br>>64<br>>64<br>8<br>32<br>>64	N/A	[45]
	B. velezensis LM2303	Cell membrane	N/A	N/A	N/A	[159]
Octapeptin B (EM-49)	B. circulans ATCC 21,656	Cell membrane	Escherichia coli SC 9251<br>Escherichia coli SC 9252<br>Escherichia coli SC 9253<br>Bacillus subtilis GSY 201	1.6 µg/mL<br>0.8 µg/mL<br>0.3 µg/mL<br>0.2 µg/mL	N/A	[160]
Aurantinin B	B. subtilis fmb60	Cell membrane	Staphylococcus aureus ATCC 25923<br>Micrococcus luteus CMCC 28001	1.56 µg/mL<br>3.12 µg/mL	Compost	[48]

			Bacillus pumilus CMCC 63202<br>Bacillus cereus ATCC 14579<br>Bacillus subtilis ATCC 168<br>Listeria monocytogenes CICC 21662<br>Enterococcus faecalis ATCC 29212<br>Pseudomonas fluorescens ATCC 49642<br>Clostridium sporogenes CICC 10385<br>Staphylococcus aureus MRSA<br>Escherichia coli ATCC 25922	0.78 µg/mL<br>0.78 µg/mL<br>1.56 µg/mL<br>1.56 µg/mL<br>1.56 µg/mL<br>1.56 µg/mL<br>0.78 µg/mL<br>6.25 µg/mL<br>>100 µg/mL		
Aurantinin C	B. subtilis fmb60	Cell membrane	Staphylococcus aureus ATCC 25923<br>Micrococcus luteus CMCC 28001<br>Bacillus pumilus CMCC 63202<br>Bacillus cereus ATCC 14579<br>Bacillus subtilis ATCC 168<br>Listeria monocytogenes CICC 21662<br>Enterococcus faecalis ATCC 29212<br>Pseudomonas fluorescens ATCC 49642<br>Clostridium sporogenes CICC 10385<br>Staphylococcus aureus MRSA<br>Escherichia coli ATCC 25922	1.56 µg/mL<br>3.12 µg/mL<br>0.78 µg/mL<br>0.78 µg/mL<br>1.56 µg/mL<br>1.56 µg/mL<br>1.56 µg/mL<br>1.56 µg/mL<br>0.78 µg/mL<br>6.25 µg/mL<br>>100 µg/mL	Compost	[48]
Aurantinin D	B. subtilis fmb60	Cell membrane	Staphylococcus aureus ATCC 25923<br>Micrococcus luteus CMCC 28001<br>Bacillus pumilus CMCC 63202<br>Bacillus cereus ATCC 14579<br>Bacillus subtilis ATCC 168<br>Listeria monocytogenes CICC 21662<br>Enterococcus faecalis ATCC 29212<br>Pseudomonas fluorescens ATCC 49642<br>Clostridium sporogenes CICC 10385<br>Staphylococcus aureus MRSA<br>Escherichia coli ATCC 25922	1.56 µg/mL<br>3.12 µg/mL<br>0.78 µg/mL<br>0.78 µg/mL<br>1.56 µg/mL<br>1.56 µg/mL<br>1.56 µg/mL<br>1.56 µg/mL<br>0.78 µg/mL<br>6.25 µg/mL<br>>100 µg/mL	Compost	[48]
Myriocin			C. albicans (ATCC 10231D-5)<br>C. glabrata (ATCC 10231D-5)<br>C. glabrata (109)<br>C. albicans (1114)<br>C. albicans (12-99)	2.0 µg/mL<br>1.0 µg/mL<br>0.5 µg/mL<br>1.0 µg/mL<br>0.25 µg/mL		[49]
	B. amyloliquefaciens LZN01	Cell membrane /Intra-cellular	N/A	N/A		[161]
Gramicidin A	B. brevis ATCC 8185	Cell membrane, DNA	Streptococcus pyogenes<br>Enterococcus faecalis<br>Streptococcus pneumoniae<br>Streptococcus agalactiae<br>Listeria monocytogenes	33 nM<br>270 nM<br>8.3 nM<br>1100 nM<br>4300 nM		[52]
Gramicidin S	N/A	Cell membrane	Enterococcus faecium 3.9µM<br>Streptococcus aureus 3.9-7.8 µM<br>Klebsiella pneumoniae 31.3 – 62.5 µM<br>Acinetobacter baumannii 15.6-62.5µM<br>Pseudomonas aeruginosa 31.3-62.5µM<br>Enterobacter cloacae 1.95 0 62.5µM	3.9 µM<br>3.9 – 7.8 µM<br>31.3 – 62.5 µM<br>15.6 – 62.5 µM<br>31.3 – 62.5 µM<br>1.98 – 62.5 µM		[55]
	B. brevis ATCC 9999	Cell membrane	N/A	N/A		[162]

	B. brevis var. G.B.	Cell membrane	N/A	N/A		[163]
Surfactin A	B. velezensis LM2303	Cell membrane	N/A	N/A	dung of wild yak inhabited Qinghai-Tibet plat-eau, China	[159]
	Bacillus subtilis PB2-L1	Cell membrane	Bacillus cereus AS1.1846<br>Staphylococcus aureus AS1.2465<br>Micrococcus luteus CMCC28000<br>Pseudomonas flurescens AS1.1802<br>Salmonella enteritidis CICC21527<br>Bacillus subtilis ATCC9943	100 μg/mL<br>50 μg/mL<br>200 μg/mL<br>400 μg/mL<br>200 μg/mL<br>100 μg/mL		[164]
	B. subtilis CMB32	Cell membrane	Colletotrichum gloeosporioides		Soil	[165]
	B. amyloliquefaciens strain FJAT-2349	Cell membrane	N/A	N/A	Soil sample from JiuHuaMountain, Anhui Province, China	[166]
	B. sp P5	Cell membrane	N/A	N/A	Puba, a regional fermentation product from cassava	[167]
	B. velezensis LHSB1	Cell membrane	N/A	N/A	Peanut seeds	[168]
	B. velezensis BvL03	Cell membrane	N/A	N/A		[169]
	B. pumilus ICVB403	Cell membrane	N/A	N/A	Copepod eggs	[170]
	Bacillus NH-100	Cell membrane	Fusarium moiliforme<br>Fusarium oxysporum<br>Fusarium solani P302<br>Fusarium solani ofio601 As5<br>Fusarium Solani SAN1077<br>Trichoderma atroviride P150907	Active<br>Active<br>Active<br>Active<br>Active<br>Active	Applied Microbiology and Biotechnology (AMB) Lab, CIIT,Islamabad	[171]
	Bacillus NH-217	Cell membrane	Fusarium moiliforme<br>Fusarium oxysporum<br>Fusarium solani P302<br>Fusarium solani ofio601 As5<br>Fusarium Solani SAN1077<br>Trichoderma atroviride P150907	Active<br>Active<br>Active<br>Active<br>Active<br>Active		[171]
	B. subtilis S1702	Cell membrane	N/A	N/A		[172]
Surfactin B	B. amyloliquefaciens HR62	Cell membrane	N/A	N/A		[173]
	B. subtilis S1702	Cell membrane	N/A	N/A		[172]
Surfactin C	Bacillus atrophaeus 176s	Cell membrane	N/A	N/A	Tortella tortuosa (Pottiaceae, Bryophyta) grown in an Austrian pine forest on limestone.	[174]
Lichenysin	B. megaterium pL6 (Project Report SC 3488)	Cell membrane	N/A	N/A	Soil, Universidade de São Paulo, São Paulo, SP, Brazil	[175]
Fengycin A	Bacillus amyloliquefaciens Q-426	Cell membrane /Cell wall, Intracellular, QS	Fusarium oxysporum f. sp. spinaciae O-27	31.25 μg/ml	Isolated from compost samples collected in the Dalianregion of China	[176]
	B. velezensis LHSB1	Cell membrane /Cell wall, Intracellular, QS	N/A	N/A		[168]
	B. subtilis HC8	Cell membrane /Cell wall, Intracellular, QS	N/A	N/A		[177]
	B. subtilis strain F-29-3	Cell membrane /Cell wall, Intracellular, QS	N/A	N/A	Isolated from potato farm	[178]
	B. megaterium pL6 (Project Report SC 3488)	Cell membrane /Cell wall, Intracellular, QS	N/A	N/A	Soil, Universidade de São Paulo, São Paulo, SP, Brazil	[175]
	B. amyloliquefaciens strain 32a	Cell membrane /Cell wall, Intracellular, QS	N/A	N/A		[179]

	B. licheniformis V9T14	Cell membrane /Cell wall, Intracellular, QS	N/A	N/A		[180]
	B. subtilis S1702	Cell membrane /Cell wall, Intracellular, QS	N/A	N/A		[172]
	B. subtilis XF-1 (CGMCC No. 2357)	Cell membrane /Cell wall, Intracellular, QS	N/A	N/A	rhizosphere soil of Chinese cabbage (Brassica pekinensis)	[181]
Fengycin B	B. velezensis LM2303	Cell membrane /Cell wall, Intracellular, QS	N/A	N/A	N/A	[159]
	Bacillus amyloliquefaciens Q-426	Cell membrane /Cell wall, Intracellular, QS	N/A	N/A		[176]
	B. subtilis strain F-29-3	Cell membrane /Cell wall, Intracellular, QS	N/A	N/A	Isolated from potato farm	[178]
	B. megaterium pL6 (Project Report SC 3488	Cell membrane /Cell wall, Intracellular, QS	N/A	N/A	Soil, Universidade de São Paulo, São Paulo, SP, Brazil	[175]
	B. mojavensis B0621A	Cell membrane /Cell wall, Intracellular, QS	Valsi mali<br>Fusarium oxysporum f. sp. cucumerinum<br>Fusarium oxysporum f. sp. vasinfectum<br>Fusarium oxysporum f. sp. vasinfectum. SF 2<br>Fusarium solani SF 130<br>Botryosphaeria berengriana f. sp. piricola<br>Botrytis cicrea<br>Rhozoctonia solani J. G. Kuhn<br>Fusarium solani<br>Rhizoctonia solani<br>Valsa ceratosperma<br>Fusarium oxysporum f. sp. Cucumis melo L.<br>Fusarium graminearum<br>Bipolaris maydis<br>Colletrotrichum orbiculare<br>Fusarium verticillioides	Active<br>Active<br>Active<br>Active<br>Active<br>Active<br>Active<br>Active<br>Active<br>Active<br>Active<br>Active<br>Active<br>Active<br>Active<br>Active	Pinctada martensii in the South China Sea	[182]
	B. licheniformis V9T14	Cell membrane /Cell wall, Intracellular, QS	N/A	N/A		[180]
	B. subtilis XF-1 (CGMCC No. 2357)	Cell membrane /Cell wall, Intracellular, QS	N/A	N/A	rhizosphere soil of Chinese cabbage (Brassica pekinensis)	[181]
	B. amyloliquefaciens strain FJAT-2349	Cell membrane /Cell wall, Intracellular, QS	N/A	N/A	Soil sample from JiuHuaMountain, Anhui Province, China	[166]
	B. amyloliquefaciens Pc3	Cell membrane /Cell wall, Intracellular, QS	N/A	N/A	Antarctic seawater	[183]
	B. amyloliquefaciens ANT1	Cell membrane /Cell wall, Intracellular, QS	N/A	N/A	Isolated from a surgical room at the St. Mary of Lourdes Clinic (Naples district, Italy	[184]
	B. subtilis HC8	Cell membrane /Cell wall, Intracellular, QS	N/A	N/A		[185]
	B. amyloliquefaciens strain 32a	Cell membrane /Cell wall, Intracellular, QS	N/A	N/A		[179]
	B. sp. MA04	Cell membrane /Cell wall, Intracellular, QS	N/A	N/A	Tomato rhizosphere soil	[186]
	B. subtilis S1702	Cell membrane /Cell wall, Intracellular, QS	N/A	N/A		[172]

Fengycin C	B. subtilis XF-1 (CGMCC No. 2357)	Cell membrane /Cell wall, Intracellular, QS	N/A	N/A	rhizosphere soil of Chinese cabbage (Brassica pekinensis)	[181]
	B. subtilis EA-CB0015	Cell membrane /Cell wall, Intracellular, QS	N/A	N/A	phyllosphere of a banana plant	[187]
	B. subtilis S1702	Cell membrane /Cell wall, Intracellular, QS	N/A	N/A		[172]
Fengycin D	B. subtilis XF-1 (CGMCC No. 2357)	Cell membrane /Cell wall, Intracellular, QS	N/A	N/A	rhizosphere soil of Chinese cabbage (Brassica pekinensis)	[181]
	B. subtilis S1702	Cell membrane /Cell wall, Intracellular, QS	N/A	N/A		[172]
Iturin A	B. velezensis LM2303	Cell membrane, Intracellular cellular processes	N/A	N/A	dung of wild yak inhabited Qinghai-Tibet plateau, China	[159]
	B. megaterium pL6 (Project Report SC 3488	Cell membrane, Intracellular cellular processes	N/A	N/A	Soil, Universidade de São Paulo, São Paulo, SP, Brazil	[175]
	B. subtilis B-3	Cell membrane, Intracellular cellular processes	Aspergillus clavatus SRRC 17<br>Aspergillus flavus SRRC 295 (nonaflatoxigenic)<br>Aspergillus flavus SRCC 2089 (aflatoxigenic)<br>Aspergillus orchaceus SRRC 335<br>Aspergillus parasiticus SRRC 1008<br>Aspergillus versicolor SRRC 108B<br>Fusarium moniliforme SRRC 1086<br>Fusarium nivale (Ferlacia) SRRC 131<br>Penicillium citrinum SRRC 1013<br>Penicillium expansum SRRC 1134<br>Penicillium italicum SRRC 1169<br>Penicillium virdicatum SRRC 217	Active<br>Active<br>Active<br>Active<br>Active<br>Active<br>Active<br>Active<br>Active<br>Active<br>Active<br>Active	N/A	[188]
	B. velezensis BvL03	Cell membrane, Intracellular cellular processes	N/A	N/A		[169]
	B. amyloliquefaciens ANT1	Cell membrane, Intracellular cellular processes	N/A	N/A	Isolated from a surgical room at the St. Mary of Lourdes Clinic (Naples district, Italy	[184]
	B. licheniformis W10	Cell membrane, Intracellular cellular processes	N/A	N/A	Isolated from a tomato rhizosphere	[189]
	B. subtilis strain KS03	Cell membrane, Intracellular cellular processes	Gloeosporium gloeosporioides	N/A	rice straw	[190]
	B. subtilis RB14-CS	Cell membrane, Intracellular cellular processes	Rhizoctonia solani,	N/A	N/A	[191]
	B. subtilis JA	Cell membrane, Intracellular cellular processes	Fusarium graminearum	N/A	Key Laboratory of Ion Beam Engineering, Chinese Academy of Sciences, Hefei 230031, Anhui province, China	[192]

	B. subtilis HC8	Cell membrane, Intra-cellular cellular processes	N/A	N/A	Giant hogweed, Heracleum sosnowskyi Manden	[177]
	B. sp. strain CS93	Cell membrane, Intra-cellular cellular processes	N/A	N/A	Pozol, Villahermosa, Tabasco, Mexico	[152]
	B. subtilis SSE4	Cell membrane, Intra-cellular cellular processes	N/A	N/A	Shrimp shells	[193]
	B. megaterium pL6 (Project Report SC 3488	Cell membrane, Intra-cellular cellular processes	N/A	N/A	Soil, Universidade de São Paulo,<br>São Paulo, SP, Brazil	[175]
	B. amyloliquefaciens PPCB004	Cell membrane, Intra-cellular cellular processes	Alternaria citri (Penz.) Mussat PPCF001<br>Botryosphaeria sp. PPAF001<br>Colletotrichum gloeosporioides (Penz.) Penz. & Sacc<br>Fusicoccum aromaticum (Sacc.) Petr. & Syd<br>Lasiodiploa theobromae (Pat.) Griffon & Maubi<br>Penicillium crustosum Thom<br>Phomopsis persea Zerova<br>Bacillus subtilis UMAF6614<br>Bacillus subtilis UMAF 6639<br>Bacillus subtilis PPCB001	Active<br>Active<br>Active<br>Active<br>Active<br>Active<br>Active<br>Active<br>Active<br>Active	Citrus fruit	[194]
	B. strain UMAF6639	Cell membrane, Intra-cellular cellular processes	Alternaria citri (Penz.) Mussat PPCF001<br>Botryosphaeria sp. PPAF001<br>Colletotrichum gloeosporioides (Penz.) Penz. & Sacc<br>Fusicoccum aromaticum (Sacc.) Petr. & Syd<br>Lasiodiploa theobromae (Pat.) Griffon & Maubi<br>Penicillium crustosum Thom<br>Phomopsis persea Zerova<br>Bacillus subtilis UMAF6614<br>Bacillus subtilis UMAF 6639<br>Bacillus subtilis PPCB001	Active<br>Active<br>Active<br>Active<br>Active<br>Active<br>Active<br>Active<br>Active<br>Active	N/A	[194]
	B. subtilis CMB32	Cell membrane, Intra-cellular cellular processes	N/A	N/A	Soil	[165]
	B. subtilis 3-10	Cell membrane, Intra-cellular cellular processes	N/A	N/A	Soil, Wuhan	[195]
	B. amyloliquefaciens S13-3	Cell membrane, Intra-cellular cellular processes	N/A	N/A	N/A	[196]
	B. amyloliquefaciens S20	Cell membrane, Intra-cellular cellular processes	Fusarium oxysporum<br>Ralstonia solanacearum	Active<br>Active	rhizosphere soil from a healthy eggplant	[197]

	B. amyloliquefaciens strain 32a	Cell membrane, Intra-cellular cellular pro-cesses	N/A	N/A	Tunisian soil sample	[179]
	B. amyloliquefaciens BAS23	Cell membrane, Intra-cellular cellular pro-cesses	Cochliobolus lunata<br>Fusarium semitectum<br>Heterodera oryzae	Active<br>Active<br>Active	Soil bacteria were isolated from rice paddy fields in Nakhon Pathom province, Thailand	[198]
	B. amyloliquefaciens L-H15	Cell membrane, Intra-cellular cellular pro-cesses	Fusarium oxysporum<br>Phytophthora capsisi<br>Rhizoctonia solani	Active<br>Active<br>Active	Cucumber seedling substrate	[199]
	B. amyloliquefaciens BUZ-14	Cell membrane, Intra-cellular cellular pro-cesses	Botrytis cinerea<br>Monilinia fructicola<br>Mandevilla laxa<br>Penicillium digitatum<br>Penicillium italicum<br>Penicillium expansum	16.9 µg/ml<br>8.5 µg/ml<br>4.1 µg/ml<br>16.9 µg/ml<br>8.5 µg/ml<br>8.5 µg/ml	surface of peach fruit from an orchard in Zara-goza	[200]
	B. amyloliquefaciens S76-3	Cell membrane, Intra-cellular cellular pro-cesses	Fusarium Graminearum	50 ug/ml	spikes of wheat grown in our own experiment fields in our university, Wuhan, China	[79]
	B. subtilis ZK0	Cell membrane, Intra-cellular cellular pro-cesses	N/A	N/A	Cotton	[201]
	B. subtilis 3057	Cell membrane, Intra-cellular cellular pro-cesses	N/A	N/A	N/A	[202]
	B. amyloliquefaciens LZ-5	Cell membrane, Intra-cellular cellular pro-cesses	Saccharomyces cerevisiae	0.76 mg/ml	Chinese honey	[203]
	B. subtilis S1702	Cell membrane, Intra-cellular cellular pro-cesses	N/A	N/A	healthy table grapes	[172]
	B. pumilus strain YSPMK11	Cell membrane, Intra-cellular cellular pro-cesses	N/A	N/A	cauliflower plants were collected from four different naturally growing agro climatic zones of Himachal Pradesh.	[204]
	B. subtilis I'1a strain	Cell membrane, Intra-cellular cellular pro-cesses	N/A	N/A	sludge of a<br>100-year-old oil refinery in Czechowice-Dziedzice (Po- land)	[205]
	B. subtilis NBRC 109107	Cell membrane, Intra-cellular cellular pro-cesses	N/A	N/A	National<br>Institute of Technology and Evaluation<br>（NITE） of Japan	[206]
	B. siamensis JFL15	Cell membrane, Intra-cellular cellular pro-cesses	N/A	N/A	isolated from the gastrointestinal tract of hair-tail	[207]
	B. subtilis strain ET-1	Cell membrane, Intra-cellular cellular pro-cesses	N/A	N/A	soil and both phenotypically and genetically identified at the microbiology laboratory of ENEA Research Centre Trisaia	[208]
	B. amyloliquefaciens DA12	Cell membrane, Intra-cellular cellular pro-cesses	Fusarium graminearum macroconidia	Active	soil in which tomatoes grew in a greenhouse in Buyeo-gun, Chungcheong	[209]

	B. amyloliquefaciens CX-20	Cell membrane, Intra-cellular cellular processes	N/A	N/A	Professor Shouwen Chen (College of Life Sciences, Hubei University, Wuhan, China).	[210]
	B. amyloliquefaciens Pc3	Cell membrane, Intra-cellular cellular processes	N/A	N/A	Antarctic seawater	[183]
	B. subtilis CCTCCM207209	Cell membrane, Intra-cellular cellular processes	N/A	N/A	Strain previously isolated from soil and stored at the China Center for Type Culture Collection (Wuhan, China)	[183]
	B. amyloliquefaciens Ba01	Cell membrane, Intra-cellular cellular processes	N/A	N/A	isolated from healthy potato tuber	[211]
	B. amyloliquefaciens strain FJAT-2349	Cell membrane, Intra-cellular cellular processes	N/A	N/A		[166]
	B. amyloliquefaciens LL3	Cell membrane, Intra-cellular cellular processes	N/A	N/A		[189]
	B. subtilis ABS-S14	Cell membrane, Intra-cellular cellular processes	N/A	N/A	isolated from soil collected from citrus groves around the south of Thailand	[212]
Bacillomycin D	Bacillus amyloliquefaciens Q-426	Cell membrane, Intra-cellular processes, siderophore	N/A	N/A	Isolated from compost samples collected in the Dalianregion of China	[176]
	B. subtilis 3057	Cell membrane, Intra-cellular cellular processes, siderophore	N/A	N/A	N/A	[202]
	B. amyloliquefaciens SQR9	Cell membrane, Intra-cellular processes, siderophore	N/A	N/A	Cucumber rhizosphere	[213]
	B. vallismortis ZZ185	Cell membrane, Intra-cellular processes, siderophore	Fusarium graminearum<br>Alternaria alternata<br>Rhizoctonia solani<br>Cryphonectria parasitica<br>Phytophthora capsici.	Active<br>Active<br>Active<br>Active<br>Active	Healthy stems of the plant Broadleaf Holly (Ilex latifolia Thunb) collected in Nanjing, China	[214]
	B. amyloliquefaciens strain SD-32	Cell membrane, Intra-cellular processes, siderophore	Podosphaera fusca	Active	Soil sample obtained in Japan	[109]
	B. amyloliquefaciens BS6	Cell membrane, Intra-cellular processes, siderophore	N/A	N/A	N/A	[202]
	B. mycoides 4079	Cell membrane, Intra-cellular processes, siderophore	N/A	N/A	N/A	[202]
	B. velezensis HN-2	Cell membrane, Intra-cellular processes, siderophore	Colletotrichum gloeosporioides	EC50 = 3.462 ug/ml	Soil	[82]
	B. velezensis FZB42	Cell membrane, Intra-cellular processes, siderophore	Fusarium oxysporum	Active		[215]

	B. sp. strain BCLRB2	Cell membrane, Intra-cellular processes, si-derophore	N/A	N/A	healthy leaves of olive tree	[216]
	B. velezensis NST6	Cell membrane, Intra-cellular processes, si-derophore	Staphylococcus aureus<br>Staphylococcus epidermidis<br>MRSA 3090	2 μg/ml<br>2 μg/ml<br>2 μg/ml	soil sample	[217]
	B. amyloliquefaciens C5	Cell membrane, Intra-cellular processes, si-derophore	N/A	N/A	roots of olive tree	[218]
	B. velezensis BvL03	Cell membrane, Intra-cellular processes, si-derophore	N/A	N/A		[169]
	B. amyloliquefaciens MTCC 10456	Cell membrane, Intra-cellular processes, si-derophore	N/A	N/A	Seaweed	[150]
	B. sp. PPM3	Cell membrane, Intra-cellular processes, si-derophore	N/A	N/A	Bacillus sp. PPM3 isolated from marine sedi-ment from the Red Sea in Hurghada, Egyp	[219]
Mycosubtilin	B. subtilis 370	Cell membrane	Mycoderma valida<br>Saccharomyces carlsbergensis, 9080<br>Rhodotorula rubra<br>Sporobolomyces roseus<br>Torula cremoris<br>Dipodascus uninucleolus<br>Hansenula anomala, 4104<br>Torulopsis delbruckii<br>Microsporum lanosum<br>Trichophyton mentagrophytes<br>Fusarium moniliforme<br>Nematospora coryli<br>Penicillium notatum<br>Chaetomium bostrychodes<br>Microsporum audouini<br>Achoriun schoenleinii<br>Cryptococcus neoformans<br>Epidermophyton inguinale<br>Sclerotinia fructicola<br>Ustilago zeae<br>Trichophyton sp.	Active<br>Active		[220]
	B. sp. PPM3		N/A	N/A		[219]
	Bacillus subtilis ATCC 6633		Saccharomyces cerevisiae<br>Yarrowia lipolytica CBS6303<br>Pichia pastoris<br>Candida albicans ATCC 10231<br>Candida albicans IHEM3742<br>Candida parapsilosis IHEM9557<br>Candida tropicalis IHEM6246<br>Candida guilliermondii IHEM 1067<br>Candida glabrata IHEM 6161<br>Candida glabrata L999	4 ug/ml<br>8 ug/ml<br>32 ug/ml<br>32 ug/ml<br>64 ug/ml<br>128 ug/ml<br>16 ug/ml<br>128 ug/ml<br>16 ug/ml<br>2 ug/ml		[221]

			Candida glabrata S53452<br>Candida glabrata H34736<br>Candida glabrata W16119<br>Cryptococcus neofarmans IHEM 3969<br>Aspergillus parasiticus IHEM4384<br>Aspergillus terreus IHEM2499<br>Aspergillus fumigatus IHEM3562	150 ug/ml<br>150 ug/ml<br>150 ug/ml<br>8 ug/ml<br>>300 ug/ml<br>>300 ug/ml<br>>300 ug/ml		
Bacillomycin L	B. amyloliquefaciens K103	Cell membrane	Rhizoctonia solani Kühn	N/A	lemon	[87]
	B. subtilis NCIB 8872	Cell membrane	N/A	N/A		[222]
	B. amyloliquefaciens SYBC H47	Cell membrane	Botryosphaeria dothidea	N/A	Peach Gummosis	[223]
Mycobacillin	B. subtilis B3	Cell membrane	Aspergillus niger strain G3Br	20 mg/ml		[94,224]
Subtilosin A	B. subtilis ATCC 19659	Cell membrane//Bio-film	N/A	N/A		[225]
	B. subtilis EMD4	Cell membrane//Bio-film	Bacillus cereus ATCC14579<br>Bacillus subtilis ATCC14593<br>Bacillus licheniformis ATCC21415<br>Bacillus thuringiensis ATCC33679<br>Enterococcus faecalis ATCC29212<br>Enterococcus faecium ATCC19953<br>Lactobacillus delbrueckii ssp. lactis ATCC4797<br>Lactobacillus caesei ssp. casei ATCC4646<br>Lactobacillus pentosus ATCC8041<br>Leuconostoc mesenteroides ATCC9135<br>Listeria monocytogenes ATCC19111<br>Pediococcus pentosaceus NRRL B-14009<br>Streptococcus thermophilus KFRI 193	Active<br>Active<br>Active<br>Active<br>Active<br>Active<br>Active<br>Active<br>Active<br>Active<br>Active<br>Active<br>Active	Soy Sauce	[226]
	B. amyloliquefaciens (EU105395)	Cell membrane//Bio-film	Micrococcus luteus ATCC 10420<br>Listeria monocytogenes Scott A<br>Gardnerella vaginalis ATCC 14018<br>Gardnerella vaginalis (clinical isolate)<br>Streptococcus agalactiae (clinical isolate)	Active<br>Active<br>Active<br>Active<br>Active		[227]
	B. amyloliquefaciens BAhja NK10	Cell membrane//Bio-film	Micrococcus luteus A1 NCIMB 8166<br>Listeria monocytogenes WSLC 1018	2.8mm<br>2.4mm	Soil	[228]
	B. tequilensis FR9	Cell membrane//Bio-film	Listeria monocytogenes MTCC 657	Active	free-range chickens GUT	[229]
	B. subtilis KATMIRA1933	Cell membrane//Bio-film	Gardnerella vaginalis<br>Listeria monocytogenes<br>Escherichia coli	6.25 µg/ml<br>125 µg/ml<br>250 µg/ml		[99]
	B. subtilis BSD-2	Cell membrane//Bio-film	N/A	N/A		[230]
	N/A	Cell membrane//Bio-film	G. vaginalis	7.2 µg/mL		[97]
	B. subtilis strain SEM-2	Cell membrane//Bio-film	N/A	N/A	silkworm excrement composting	[231]
Zwittermicin A	B. thuringiensis HD-1	Intracellular processes	Erwinia herbicola L S005<br>Escherichia coli K37	60 µg/ml<br>100 µg/ml		[100]

			Bacillus subtilis 168<br>Staphylococcus aureus 3001	120 µg/ml<br>200 µg/ml		
	B. cereus UW85	Intracellular processes	N/A	N/A	Root of a field-grown alfalfa plant from Arlington, WI	[23]
	B. amyloliquefaciens BS6	Intracellular processes	N/A	N/A		[202]
	B. mycoides S	Intracellular processes	N/A	N/A		[202]
	B. thuringiensis BS8	Intracellular processes	N/A	N/A		[202]
Difficidin	B. subtilis ATCC 39320	Intracellular processes	Escherichia coli MB 4827	35 µg/ml		[103]
	B. velezensis LM2303	Intracellular processes	N/A	N/A	dung of wild yak inhabited Qinghai-Tibet plateau, China	[159]
	B. velezensis LDO2	Intracellular processes	N/A	N/A	peanut	[232]
	B. velezensis FZB42	Intracellular processes	N/A	N/A		[158]
	B. velezensis V4	Intracellular processes	N/A	N/A		[233]
	B. amyloliquefaciens subsp. plantarum B9601-Y2	Intracellular processes	N/A	N/A		[156]
	B. subtilis S499	Intracellular processes	N/A	N/A		[157]
Sublancin (Lantibiotic)	B. subtilis 168	Intracellular processes	MRSA	15 µM		[105]
	Bacillus subtilis A52	Intracellular processes	Staphylococcus aureus MTCC 1433<br>Bacillus subtilis MTCC 121<br>Bacillus cereus MTCC 430<br>Bacillus coagulans MTCC 492<br>Staphylococcus pyogenes MTCC 1928<br>Streptococcus anginosus MTCC 1929<br>Streptococcus oralis MTCC 2696<br>Streptococcus mutans MTCC 497<br>Micrococcus luteus MTCC 106	10 ug/ml<br>5 ug/ml<br>5 ug/ml<br>8 ug/ml<br>2 ug/ml<br>6 ug/ml<br>8 ug/ml<br>10 ug/ml<br>2 ug/ml		[234]
Amicoumacin A	B. subtilis B1779	Intracellular processes	Bacllus subtillis<br>Staphylococcus Aureus<br>Loktanella Hongkongensis	18.87 µM<br>18.87 µM<br>1.18 µM	Marine sediment at depth of 1000 m in the Red Sea	[235]
			Bacillus subtilis 1779<br>Staphylococcus aureus UST950701-005<br>Methicillin-resistant Staphylococcus aureus ATCC43300	20 µM<br>5.0 µM<br>4.0 µM		[236]
	B. pumilus BN-103	Intracellular processes	N/A	N/A		[237]
	B. subtilis BSXE-1601	Intracellular processes	Vibrio vulnificus S01P2<br>Vibrio harveyi SRTT9<br>Vibrio alginolyticus AR-1<br>Vibrio parahaemolyticus 20130629002S01<br>Pseudoalteromonas sp. LPE40<br>Shewanella marisflavi AP629<br>Streptococcus iniae iniae NUF849<br>Aeromonas hydrophila AP40301<br>Edwardsiella tarda HC01090721<br>Vibrio splendidus BSD11	1.25 ug/ml<br>1.25 ug/ml<br>1.25 ug/ml<br>1.25 ug/ml<br>1.25 ug/ml<br>1.25 ug/ml<br>10 ug/ml<br>>10 ug/ml<br>>10 ug/ml<br>>10 ug/ml		[238]
Prumycin	B. amyloliquefaciens SD-32	Intracellular processes	N/A	N/A		[109]
			Staphyloccus aureus FDA 209P<br>Bacillus subtilis PCI 219	>100<br>>100		[111]

			Sarcina lutea PCI 1001	3.12		
			Salmonella typhimurium	>100		
			Escherichia coli NIHJ	100		
			Pseudomonas aeruginosa P-3	>100		
			Klebsiella pneumoniae	>100		
			Shigella sonnei	100		
			Vibrio comma 904	12.5		
			Proteus vulgaris OX-19	>100		
			Mycobacterium ATCC 607	>100		
			Xanthomonas oryzae	50		
			Xanthomonas citri	>100		
			Alternaria mali	>100		
			Alternaria kikuchiana	>100		
			Alternaria japonica	50		
			Sclerotinia cinerea	12.5		
			Sclerotinia sclerotiorum	1.56		
			Botorytis fabae	6.25		
			Botorytis cinerea	6.25		
			Botorytis cyptoneriae	>100		
			Cochliobolus miyabeanus	50		
			Collectotrichum lagenarium	50		
			Candida albicans	>100		
			Saccharomyces cerevisiae	>100		
			Cryptococcus neoformans	>100		
			Hormodendrum pedrosoi	25		
			Fusarium moniliforme USDA 1004-1	>100		
			Penicillium notatum	>100		
			Trichophyton rubrum	100		
			Trichophyton mentagrophytes	100		
			Trichosporon beigellii	>100		
			Glasosporium laeticolotor	50		
			Glomerella cingulate	100		
			Ophiobolus miyabeanus	25		
			Aspergillus niger	>100		
Thiocillin	B. cereus (strain ATCC 14579)	Intracellular processes	N/A	N/A		[239]
		Intracellular processes	Staphylococcus aureus ATCC 29213	2 ug/ml		[240]
			Staphylococcus aureus 1974149	2 ug/ml		
			Staphylococcus aureus 1974148	2 ug/ml		
			Enterococcus faecalis 1674621	0.5 ug/ml		
			Enterococcus faecalis 1674614	0.5 ug/ml		
			Bacillus subtilis ATCC 6633	4 ug/ml		
			Streptococcus pyogenes 1744264	0.5 ug/ml		
Hetiamacin E	B. subtilis PJS	Intracellular processes	Staphylococcus epidermidis ATCC 12228 (MSSE)	2 µg/mL		[114]
			Staphylococcus epidermidis 16-4 (MSSE)	4 µg/mL		
			Staphylococcus epidermidis 16-5 (MRSE)	4 µg/mL		
			Staphylococcus aureus ATCC 29213 (MSSA)	8 µg/mL		
			Staphylococcus aureus ATCC 33591 (MRSA)	16 µg/mL		

			Enterococcus faecium ATCC 700221 (VRE)<br>Escherichia coli ATCC 25922<br>Pseudomonas aeruginosa PAO1<br>Acinetobacter baumannii ATCC 19606<br>Shigella flexneri ATCC 12022	64 μg/mL<br>64 μg/mL<br>64 μg/mL<br>64 μg/mL<br>64 μg/mL		
Hetiamacin F	B. subtilis PJS	Intracellular processes	Staphylococcus epidermidis ATCC 12228 (MSSE)<br>Staphylococcus epidermidis 16-4 (MSSE)<br>Staphylococcus epidermidis 16-5 (MRSE)<br>Staphylococcus aureus ATCC 29213 (MSSA)<br>Staphylococcus aureus ATCC 33591 (MRSA)<br>Enterococcus faecium ATCC 700221 (VRE)<br>Escherichia coli ATCC 25922<br>Pseudomonas aeruginosa PAO1<br>Acinetobacter baumannii ATCC 19606<br>Shigella flexneri ATCC 12022	32 μg/mL<br>32 μg/mL<br>32 μg/mL<br>>32 μg/mL<br>>32 μg/mL<br>>32 μg/mL<br>>32 μg/mL<br>32 μg/mL<br>32 μg/mL<br>>32 μg/mL		[114]
Rhizocticin A	B. cabrialesii TE3	Intracellular processes	N/A	N/A		[241]
	B. subtilis NBRC3134	Intracellular processes	N/A	N/A		[242]
	B. subtilis ATCC 6633	Intracellular processes	Budding and filamentous fungi (Not available)<br>Caenorhabditis elegans	N/A		[116]
Macrolactin N	B. subtilis AT29	Intracellular processes	Staphylococcus aureus PDF<br>Escherichia coli<br>Staphylococcus aureus<br>Bacillus subtilis	IC50 = 7.5 μg/mL<br>MIC = 100 μg/mL<br>MIC50 = 100 μg/mL<br>MIC50 = 100 μg/mL		[117]
Azoxybacillin	B. cereus NR2991	Intracellular processes	Aspergillus fumigatus<br>Trichophyton mentagrophytes<br>Candida albicans	IC80 = 0.71 ~ 1.3 μg/mL<br>IC80 = 0.03~ 0.04 μg/mL<br>Ic80: 4.2~ 5.8 μg/mL		[119]
Stigmatellin Y	B. subtilis BR4	Quorum sensing	Pseudomonas aeruginosa (ATCC 27853)	N/A		[124]
Bacillaene	N/A	Quorum sensing	Escherichia coli K10<br>Escherichia coli SC10909<br>Escherichia coli SC10857<br>Escherichia coli SC10896<br>Escherichia coli BAS2006<br>Escherichia coli BAS847	37.0 μg/mL<br>0.09 μg/mL<br>5.0 μg/mL<br>0.6 μg/mL<br>0.6 μg/mL<br>0.5 μg/mL		[243]
	B. velezensis LM2303	Quorum sensing	N/A	N/A	dung of wild yak inhabited Qinghai-Tibet plateau, China	[159]
	B. subtilis XF-1 (CGMCC No. 2357)	Quorum sensing	N/A	N/A	rhizosphere soil of Chinese cabbage (Brassica pekinensis)	[43]

	B. amyloliquefaciens C-1	Quorum sensing	N/A	N/A		[244]
	B. amyloliquefaciens DH-4	Quorum sensing	N/A	N/A		[245]
	B. subtilis 3610	Quorum sensing	N/A	N/A		[246]
	B. velezensis FZB42	Quorum sensing	N/A	N/A		[158]
	B. amyloliquefaciens ssp. plantarum F11	Quorum sensing	N/A	N/A		[247]
	B. subtilis strain SEM-2	Quorum sensing	N/A	N/A		[232]
	B. subtilis ATCC 55422	Quorum sensing	N/A	N/A		[248]
	Bacillus amyloliquefaciens subsp. plantarum B9601-Y2	Quorum sensing	N/A	N/A	wheat rhizosphere	[156]
	B. amyloliquefaciens LL3	Quorum sensing	N/A	N/A		[189]
Bacillibactin	B. amyloliquefaciens MBI600		Pseudomonas syringae pv. Tomato<br>V. dahliae 70wt-r1<br>R. solani AG2-1<br>A. flavus CBS128202<br>Fusarium oxysporum f. sp. radices – lyco-persici – FRL1	N/A		[249]
	B. velezensis LM2303	Quorum sensing	N/A	N/A	dung of wild yak inhabited Qinghai-Tibet plat-eau, China	[159]
	B. subtilis S499		N/A	N/A		[157]
	B. subtilis BSD-2	Quorum sensing	N/A	N/A		[231]
	B. siamensis JFL15	Quorum sensing	N/A	N/A	isolated from the gastrointestinal tract of hair-tail	[207]
	B. subtilis strain SEM-2	Quorum sensing	N/A	N/A	silkworm excrement composting	[231]
Itoic Acid	B. subtilis B-1471	Quorum sensing	N/A	N/A		[250]
		Quorum sensing	Saccharomyces cerevisiae	N/A		[251]
Schizokinen	B. megaterium ATCC 19213	Quorum sensing	N/A	N/A		[128]
		Quorum sensing	Saccharomyces cerevisiae	N/A		[251]

ZoI = Zone of inhibition.
